# Retrohoming of a Mobile Group II Intron in Human Cells Suggests How Eukaryotes Limit Group II Intron Proliferation

**DOI:** 10.1371/journal.pgen.1005422

**Published:** 2015-08-04

**Authors:** David M. Truong, F. Curtis Hewitt, Joseph H. Hanson, Xiaoxia Cui, Alan M. Lambowitz

**Affiliations:** Institute for Cellular and Molecular Biology, Department of Molecular Biosciences, The University of Texas at Austin, Austin, Texas, United States of America; The University of North Carolina at Chapel Hill, UNITED STATES

## Abstract

Mobile bacterial group II introns are evolutionary ancestors of spliceosomal introns and retroelements in eukaryotes. They consist of an autocatalytic intron RNA (a “ribozyme”) and an intron-encoded reverse transcriptase, which function together to promote intron integration into new DNA sites by a mechanism termed “retrohoming”. Although mobile group II introns splice and retrohome efficiently in bacteria, all examined thus far function inefficiently in eukaryotes, where their ribozyme activity is limited by low Mg^2+^ concentrations, and intron-containing transcripts are subject to nonsense-mediated decay (NMD) and translational repression. Here, by using RNA polymerase II to express a humanized group II intron reverse transcriptase and T7 RNA polymerase to express intron transcripts resistant to NMD, we find that simply supplementing culture medium with Mg^2+^ induces the *Lactococcus lactis* Ll.LtrB intron to retrohome into plasmid and chromosomal sites, the latter at frequencies up to ~0.1%, in viable HEK-293 cells. Surprisingly, under these conditions, the Ll.LtrB intron reverse transcriptase is required for retrohoming but not for RNA splicing as in bacteria. By using a genetic assay for *in vivo* selections combined with deep sequencing, we identified intron RNA mutations that enhance retrohoming in human cells, but <4-fold and not without added Mg^2+^. Further, the selected mutations lie outside the ribozyme catalytic core, which appears not readily modified to function efficiently at low Mg^2+^ concentrations. Our results reveal differences between group II intron retrohoming in human cells and bacteria and suggest constraints on critical nucleotide residues of the ribozyme core that limit how much group II intron retrohoming in eukaryotes can be enhanced. These findings have implications for group II intron use for gene targeting in eukaryotes and suggest how differences in intracellular Mg^2+^ concentrations between bacteria and eukarya may have impacted the evolution of introns and gene expression mechanisms.

## Author Summary

Mobile group II introns are bacterial retrotransposons that are evolutionary ancestors of spliceosomal introns and retroelements in eukaryotes. They consist of an autocatalytic intron RNA (a ribozyme) and an intron-encoded reverse transcriptase, which together promote intron mobility to new DNA sites by a mechanism called retrohoming. Although found in bacteria, archaea and eukaryotic organelles, group II introns are absent from eukaryotic nuclear genomes, where host defenses impede their expression and lower intracellular Mg^2+^ concentrations limit their ribozyme activity. Here, we developed a mobile group II intron expression system that bypasses expression barriers and show that simply adding Mg^2+^ to culture medium enables group II intron retrohoming into plasmid and chromosomal target sites in human cells at appreciable frequencies. Genetic selections and deep sequencing identified intron RNA mutations that moderately enhance retrohoming in human cells, but not without added Mg^2+^. Thus, low Mg^2+^ concentrations in human cells are a natural barrier to efficient retrohoming that is not readily overcome by mutational variation and selection. Our results have implications for group II intron use for gene targeting in higher organisms and highlight the impact of different intracellular environments on intron evolution and gene expression mechanisms in bacteria and eukarya.

## Introduction

Mobile group II introns are retrotransposons that also function as self-splicing introns [1]. They are found in bacteria, archaea, and in the bacterial endosymbiont-derived mitochondrial and chloroplast genomes of some eukaryotes, particularly fungi and plants [2]. Despite their prokaryotic origin, mobile group II introns are believed to have strongly impacted eukaryotic nuclear genomes as evolutionary ancestors of spliceosomal introns, the spliceosome, LINEs and other non-LTR retrotransposons, and telomerase [3,4]. Mobile group II introns insert into new DNA sites by a ribozyme-based site-specific DNA integration mechanism called retrohoming, which is thought to have enabled mobile group II introns or their close relatives to proliferate within the nuclear genomes of early eukaryotes before evolving into spliceosomal introns [4,5]. In addition to its evolutionary significance, retrohoming underlies the use of group II introns as gene targeting vectors (“targetrons”), which use intron RNA/DNA target site base-pairing interactions to achieve high and programmable DNA target specificity [6–8]. Targetrons are widely used for gene targeting in bacteria, where retrohoming frequencies are high enough to identify targeting events by colony PCR screening without using genetic markers [9]. By contrast, mobile group II introns and targetrons derived from them function inefficiently in eukaryotes [10–12], and group II introns appear to be completely absent from the nuclear genomes of present-day eukaryotes [13]. The reasons for the different behavior of group II introns in prokaryotes and eukaryotes and factors that dictated their conversion into spliceosomal introns and exclusion from eukaryotic nuclear genomes remain incompletely understood.

Mobile group II introns consist of a catalytically active intron RNA (a ribozyme) and an intron-encoded reverse transcriptase (RT), which function together to promote both RNA splicing and retrohoming [1]. The intron RNA catalyzes its own splicing from a precursor RNA via two sequential transesterification reactions that result in ligated exons and an excised intron lariat RNA, identical to the splicing reaction mechanism used by spliceosomal introns in higher organisms [4,14]. To catalyze splicing, the intron RNA folds into a conserved tertiary structure that consists of six interacting secondary structure domains (DI-DVI), with three distinct structural subclasses of group II introns, IIA, IIB, and IIC, distinguished by secondary and tertiary structure features [1]. This folded RNA forms a ribozyme active site that includes nucleotide residues highly conserved in all three group II intron subclasses and utilizes site-specifically bound Mg^2+^ ions to catalyze RNA splicing and reverse splicing reactions [15–17]. The group II intron RT contributes to splicing by binding to the intron RNA and promoting formation of this catalytically active RNA structure [18–20]. After splicing, the RT remains bound to the excised intron lariat RNA in a ribonucleoprotein particle (RNP) that initiates retrohoming by recognizing a DNA target site [21]. DNA target site recognition is primarily by base pairing of sequence elements within the intron RNA to DNA sequences spanning the intron-insertion site, with only a small contribution of the group II intron RT, which helps promote local DNA melting [22]. The intron RNA then uses its ribozyme activity to insert directly into the retrohoming site, where it is reverse transcribed by the intron-encoded RT into an intron cDNA that is integrated into the genome by host enzymes [5,23–26].

Early findings that group II introns use the same splicing reaction mechanism as spliceosomal introns and that some organellar group II introns have been fragmented by DNA recombination into two or three unlinked segments that reassociate to promote RNA splicing suggested an evolutionary relationship to spliceosomal introns and a possible evolutionary origin for present-day snRNAs [27]. Recently, these hypotheses have been strongly supported by group II intron RNA crystal structures and biochemical studies, which demonstrate striking structural and functional similarities between group II intron domains and three key snRNAs (U4, U5, and U6) that comprise the catalytic core of the spliceosome [17,28–31]. The similarities include identical RNA-catalyzed splicing reactions based on similarly positioned catalytic Mg^2+^ ions at the RNA active site [15,16,30,31]. Moreover, recent structural and bioinformatic studies indicate that the conserved spliceosomal core protein Prp8 was derived from a group II intron-like RT and functions similarly as a structural scaffold for an RNA catalytic core [32,33]. Considered together with the phylogenetic distribution of group II introns, these findings support a scenario in which mobile group II introns entered ancestral eukaryotes along with bacterial endosymbionts that gave rise to mitochondria, invaded the nucleus, proliferated to high copy number, and then degenerated into snRNAs [34]. Further, this proliferation of introns in eukaryotic nuclear genes is hypothesized to have been a major driving force for the evolution of eukaryotes themselves, including for features such as (i) the nuclear membrane to separate transcription and splicing from translation, thereby limiting mistranslation of intron-containing RNAs; (ii) nonsense-mediated decay (NMD) to degrade unspliced or misspliced intron-containing transcripts that escape to the cytosol; and (iii) large-scale alternative splicing, enabling greater organismal complexity within constraints on genome size [3].

Several factors have been identified that limit group II intron function and their ability to propagate in eukaryotes. First, studies in *Saccharomyces cerevisiae* showed that RNA polymerase II (Pol II) transcripts containing *the Lactococcus lactis* Ll.LtrB group II intron, which belongs to subgroup IIA, are subject to both NMD and translational repression, leading to their accumulation in cytoplasmic foci [11]. This translational repression appears to reflect strong intermolecular base-pairing interactions between the ligated-exon junction sequence in the spliced mRNA and the excised intron or intron-containing precursor RNAs, which may impede translating ribosomes and/or target the RNA for degradation [35]. A second factor affecting group II intron propagation in eukaryotes appears to be suboptimal intracellular Mg^2+^ concentrations, which limit group II intron ribozyme activity [10]. Group II intron splicing and retrohoming both require relatively high Mg^2+^ concentrations compared to other cellular processes, and Mg^2+^ concentrations appear to be significantly lower in eukaryotes than in bacteria [10,36–38]. Studies of *S*. *cerevisiae* mtDNA introns by Schweyen and coworkers showed that mutations in a mitochondrial Mg^2+^ transporter inhibit the splicing of all four group II introns, including both subgroup IIA and IIB introns, while having minimal effect on the transcription or splicing of group I introns, which use a different ribozyme-based splicing mechanism that is less sensitive to Mg^2+^ concentration [37]. Further, microinjection assays in *Xenopus laevis* oocyte nuclei or *Drosophila* and zebrafish embryos showed that *in vitro* reconstituted Ll.LtrB group II intron RNPs could retrohome efficiently into plasmid target sites only if additional Mg^2+^ was co-injected with the plasmid DNA [10]. An attempt to overcome this limitation in human cells by using an algal mitochondrial group IIB intron (Pl.LSU/2) that self-splices at physiological Mg^2+^ concentrations *in vitro*, was unsuccessful [12], perhaps because efficient self-splicing of this intron at low Mg^2+^ concentrations requires the presence of 1 M NH_4_Cl [39]. Recently, we selected variants of the Ll.LtrB group II intron with mutations in catalytic core domain V (DV) that retrohome 10- to 20-fold more efficiently than the wild-type intron in a Mg^2+^-deficient *E*. *coli* strain [36]. These findings suggested that it might be possible to overcome the high Mg^2+^ requirement that prevents efficient group II intron retrohoming in eukaryotes by mutations at a few critical sites within the intron RNA.

Here, we developed a mobile group II intron expression system for human cells that utilizes an Ll.LtrB group II intron RNA expressed by using T7 RNA polymerase (T7 RNAP) to overcome NMD and a separately expressed human codon-optimized group II intron RT. By using this expression system, we found that simply supplementing the cell culture medium with 20–80 mM Mg^2+^ enables the Ll.LtrB intron to retrohome into plasmid and genomic target sites, the latter at frequencies of up to ~ 0.1%, in viable human cells. Further, we performed multiple rounds of *in vivo* selection of the intron ribozyme, analyzed the fitness landscape using Pacific Biosciences deep sequencing, and identified positively selected mutations that were used for synthetic shuffling to generate Ll.LtrB variants that show enhanced retrohoming in human cells. However, the maximum enhancement was <4-fold and still required extra Mg^2+^ in the culture medium. These findings indicate that low Mg^2+^ concentrations constitute a natural barrier to efficient retrohoming in eukaryotes that is not readily overcome by mutational variation and selection, and they have implications for the use of group II introns for gene targeting in higher organisms and the evolution of introns and gene expression mechanisms.

## Results

### A group II intron expression system for human cells

**Fig 1:**
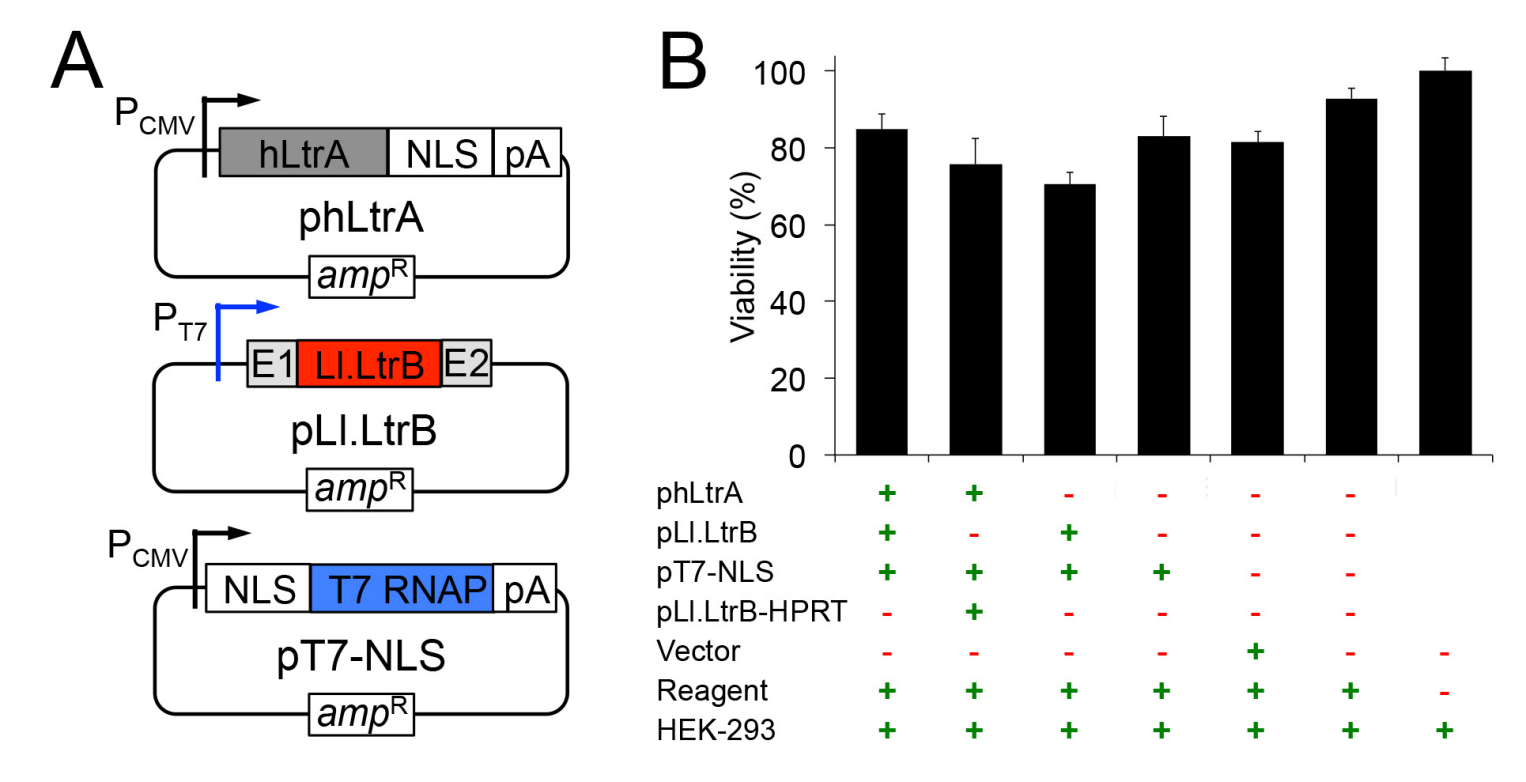
Plasmids used for expressing the mobile Ll.LtrB group II intron in human cells and their effect on cell viability. (A) Mobile group II intron expression plasmids. phLtrA uses a CMV promoter to express a humanized LtrA protein (hLtrA) with a C-terminal SV40 NLS followed by a human growth hormone polyadenylation signal (pA). pLl.LtrB uses a minimal T7 promoter to express the Ll.LtrB-ΔORF intron with flanking 5’ and 3’ *ltrB* exons (E1 and E2, respectively). pT7-NLS uses a CMV promoter to express T7 RNAP with an N-terminal SV40 NLS followed by the same polyadenylation signal as above. (B) Cytotoxicity assays. HEK-293 cells were transfected with the indicated plasmids. After 48 h in culture, luciferase activity was measured as an indicator of total cellular ATP content and cell viability by using a CellTiter-Glo direct lysis kit. Plasmid pLl.LtrB-HPRT expresses an Ll.LtrB intron targeted to the mouse *hprt* gene [45], the vector is pBluescript and the reagent is Lipofectamine 2000. The bar graph shows the average for three separate transfections with the error bars indicating the SEM.

The mobile group II intron expression system that we developed for human cells consists of three plasmids (Fig 1A). The first plasmid, denoted phLtrA, expresses the Ll.LtrB group II intron RT (denoted LtrA protein) with humanized codon usage and a C-terminal SV40 nuclear localization sequence (NLS) (NCBI Genbank, accession number KP851976)[40]. The humanized LtrA ORF is expressed from a constitutive RNA polymerase II (Pol II) promoter, the cytomegalovirus immediate early (CMV) promoter [41], and is followed by a polyadenylation signal (pA). An early version of this plasmid, phLtrA1, has a small artificial spliceosomal intron (IVS) inserted after the initiation codon [42] that was later found to be unnecessary for expression of hLtrA. The second plasmid, pLl.LtrB, uses a phage T7 promoter to express the Ll.LtrB intron with the LtrA ORF deleted (denoted Ll.LtrB-ΔORF) and short flanking 5’- and 3’-exon sequences (denoted E1 and E2, respectively). Finally, the third plasmid, pT7-NLS, expresses phage T7 RNAP with a fused N-terminal SV40 NLS driven by a CMV promoter. Previous work showed that T7 RNAP can produce high levels of uncapped, non-polyadenylated transcripts in human cells [43] and that its subcellular localization can be controlled, with nearly complete cytoplasmic or nuclear localization when expressed without or with an appended SV40 NLS, respectively [44]. The group II intron expression plasmids were not toxic when transfected by themselves or together into HEK-293 cells (Fig 1B).

### Human-codon optimized LtrA is expressed efficiently in human cells, has RT activity, and is localized to the nucleus by an NLS

**Fig 2:**
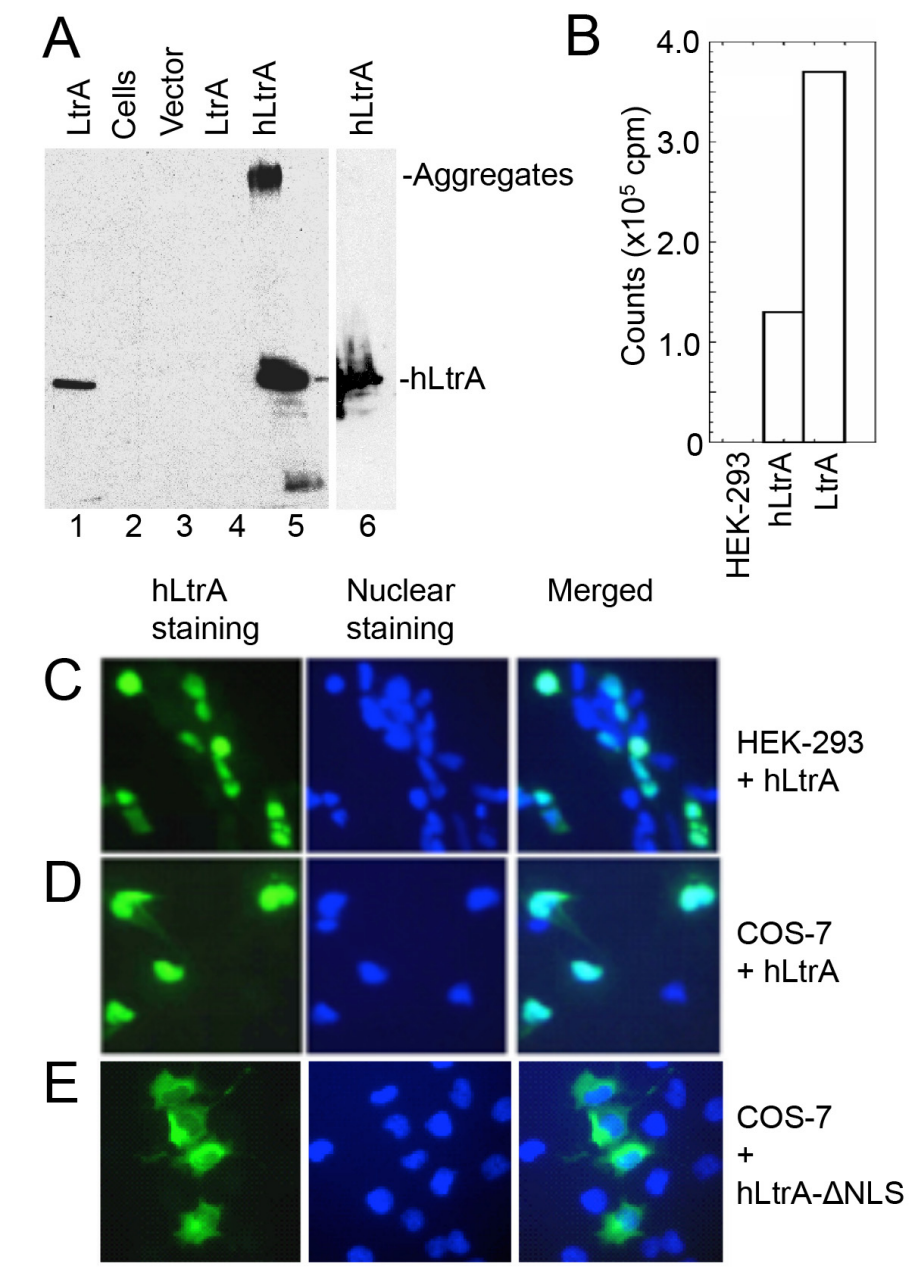
Human codon-optimized LtrA protein (hLtrA) with an SV40-NLS expressed in human cells has reverse transcriptase activity and localizes to the nucleus. (A) Immunoblots showing expression of codon-optimized hLtrA or non-codon optimized LtrA in transiently transfected HEK-293 cells after 48 h. After dissolving cells in SDS-containing gel loading buffer and boiling for 5 min, insoluble material was pelleted in a microfuge and the supernatant was loaded on the gel: Lanes: (1) LtrA purified from *E*. *coli*; (2) untransfected cells; (3) cells transfected with empty vector (pKSBluescript); (4) cells transfected with pLtrA expressing wild-type LtrA with non-optimized codons; (5) cells transfected with phLtrA1 expressing human codon-optimized hLtrA. (6) Insoluble material from cells transfected with phLtrA1 resuspended in SDS-containing gel loading buffer and boiled for an additional 5 min to dissolve aggregated hLtrA. (B) Reverse transcriptase assay. RT activity in nuclear lysates from HEK-293 cells, HEK-293 cells transfected with phLtrA1, and purified bacterially-expressed LtrA protein was assayed with a substrate comprised of a 0.9-kb Ll.LtrB-ΔORF RNA and flanking exons with a DNA primer (E2+10) annealed to the downstream exon [47]. (C-E) Immunofluorescence assay of hLtrA localization in HEK-293 and COS-7 cells. The assay was done by using an anti-LtrA antibody with a secondary antibody conjugated with fluorescein isothiocyanate (FITC) at 48 h after transfection of phLtrA1 or phLtrA1-ΔNLS. Left panels, hLtrA immunofluorescence; middle panels, nuclei stained with Hoechst dye; right panels, merge of left and middle panels.

We first compared expression of the LtrA protein with and without human optimized codons in HEK-293 cells. As shown in Fig 2A, the plasmid expressing the human-codon optimized LtrA ORF produced hLtrA protein that was readily detected by immunoblotting (lane 5 and 6), whereas an identical plasmid with a native non-codon-optimized LtrA ORF produced no detectable LtrA protein (lane 4). Further, nuclear lysates from HEK-293 cells transfected with the plasmid expressing hLtrA but not untransfected cells showed a high level of RT activity with a substrate that is efficiently used by purified LtrA protein (Ll.LtrB/E2+10 RNA; an Ll.LtrB intron-containing transcript with a DNA primer annealed downstream of the intron; Fig 2B). Immunofluorescence microscopy showed that the hLtrA expressed with a C-terminal NLS localized to the nucleus in HEK-293 cells and COS-7 cells, whereas hLtrA expressed without an added NLS (ΔNLS) localized to the cytoplasm (Fig 2C–2E). The requirements of LtrA for codon optimization and addition of an NLS to localize to the nucleus differ from recent findings for the *Sinorhizobium meliloti* RmInt1 group II intron RT, which does not require codon optimization and localizes to nucleoli in *Arabidopsis thaliana* protoplasts without an added NLS [46]. Together, our results show that optimization toward human codon usage overcomes a barrier to the expression of the Ll.LtrB group II intron RT in eukaryotes and that an appended NLS is required to localize this protein to the nucleus.

### An RNA Pol II transcript containing the Ll.LtrB intron is subject to NMD in human cells

**Fig 3:**
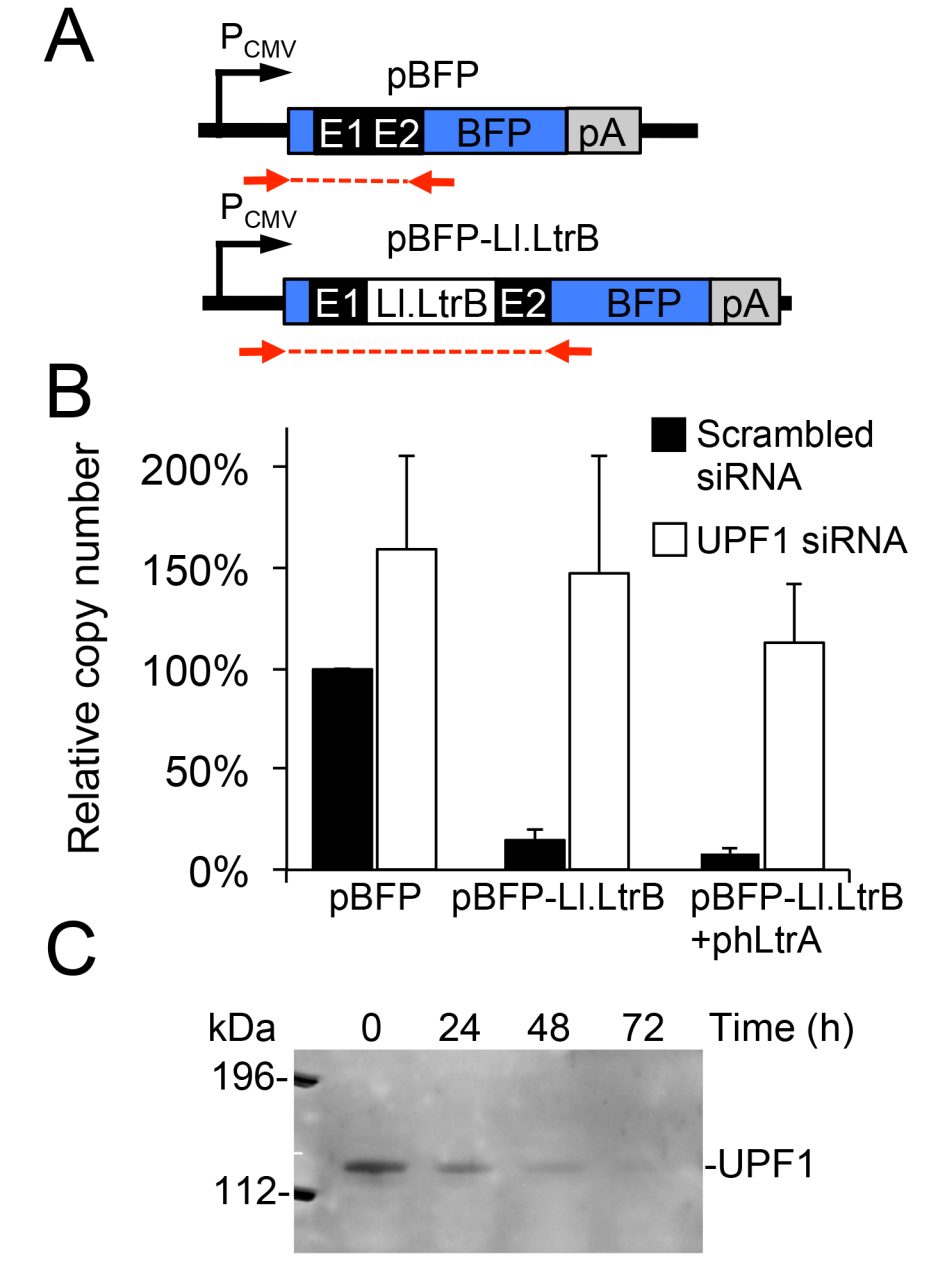
A Pol II transcript containing the Ll.LtrB intron is subject to nonsense-mediated decay in human cells. (A) Diagram of plasmid-borne P_CMV_ transcription cassettes with or without the Ll.LtrB intron and short flanking exons inserted directly after the start codon of BFP. Red arrows indicate primers used for RT-qPCR analysis of transcript levels. (B) RNAi knockdown of UPF1. HeLa cells were pretreated for 24 h with either a scrambled siRNA (black) or UPF1 siRNA (white) to inhibit NMD, and then transfected with BFP expression plasmids with or without the Ll.LtrB intron and short flanking exons inserted directly after the BFP start codon. At 48 h after transfection of the plasmids, the number of transcripts per cell was measured by RT-qPCR, and normalized to that of the pBFP transcript in the presence of the scrambled control siRNA assayed in parallel. The bar graphs show the average ± the SD for two or three replicates for each condition. (C) Immunoblot showing knockdown of the NMD protein UPF1 at 72 h after transfection of the UPF1 siRNA corresponding to the time at which BFP transcript levels were measured. Equal amounts of cellular proteins were loaded in each lane. This immunoblot control was done twice with similar results. Abbreviations: E1 and E2, 5’ and 3’ *ltrB* exons, respectively; pA, polyadenylation signal; PCMV, cytomegalovirus immediate-early promoter.

Previous studies in *S*. *cerevisiae* showed that RNA polymerase II (Pol II) transcripts containing the Ll.LtrB intron are subject to both NMD and translational repression [11]. To determine the effect of NMD on group II intron-containing transcripts in human cells, we constructed plasmids that use a CMV (Pol II) promoter to express blue fluorescent protein (BFP) with or without the Ll.LtrB-ΔORF intron and short flanking exon sequences (E1 and E2) inserted directly after the BFP start codon (Fig 3A). We transfected the plasmids into HeLa cells that were pre-treated with siRNAs targeted against *UPF1* mRNA, which encodes an essential component of the NMD complex [48], or a scrambled siRNA control, and then quantified BFP transcript levels by RT-qPCR at 48 h after plasmid transfection. As shown in Fig 3B, transcript levels for the uninterrupted BFP ORF remained high in the presence of both the UPF1 and scrambled control siRNA, with little if any significant effect of NMD knockdown. By contrast, the inclusion of the Ll.LtrB intron in the BFP ORF led to a strong decrease in transcript level in the presence of the control siRNA, but not in the presence of the UPF1 siRNA to block NMD, irrespective of co-expression of the LtrA protein. UPF1 knockdown was confirmed by immunoblotting (Fig 3C). These findings indicate that the NMD pathway degrades Pol II transcripts containing the Ll.LtrB intron in human cells as it does in *S*. *cerevisiae* [11].

### A T7 RNA polymerase-transcript containing the Ll.LtrB intron is not subject to NMD and and is spliced after addition of Mg^2+^ to the culture medium

**Fig 4:**
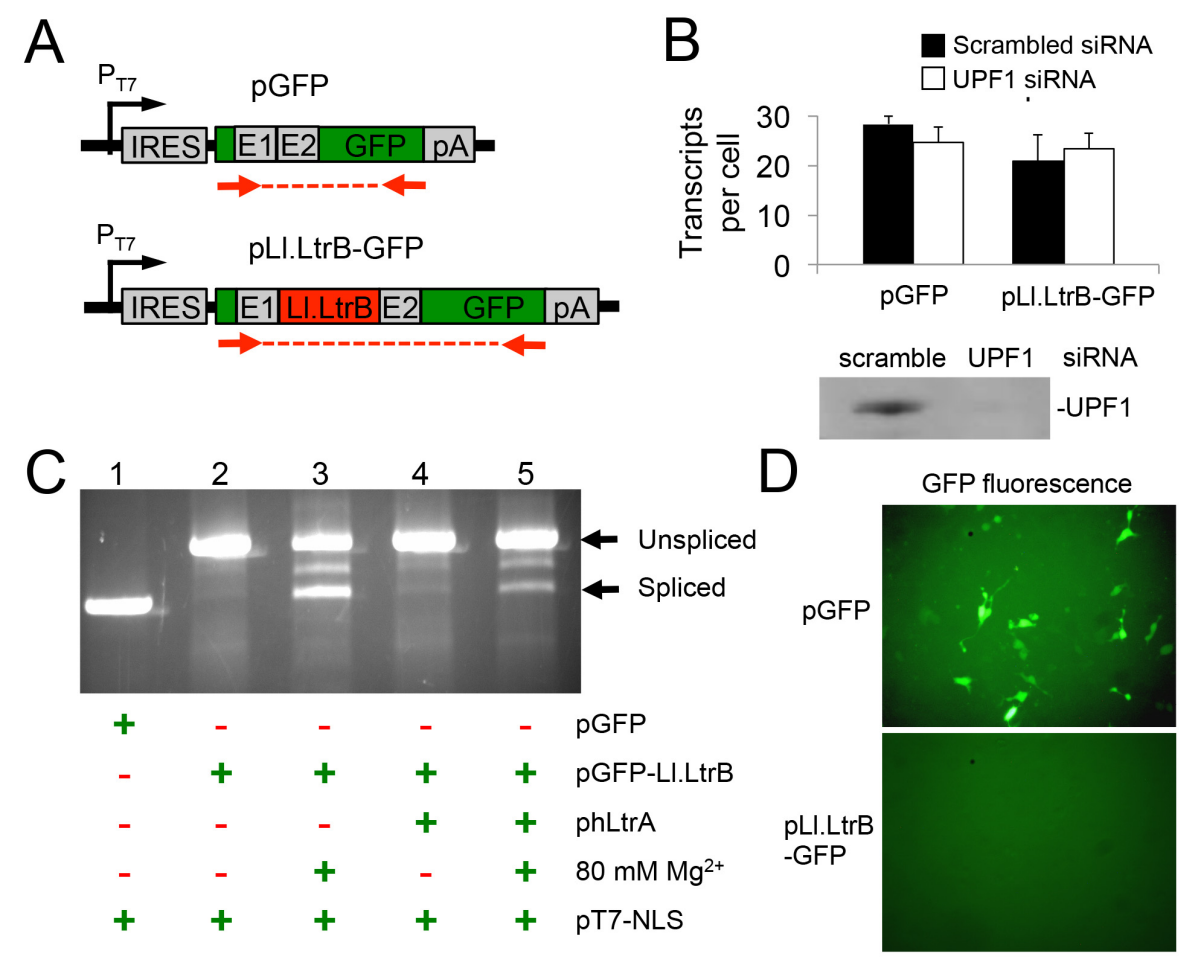
A T7 RNAP transcript containing the Ll.LtrB intron is not degraded by nonsense mediated decay in human cells and can be spliced after addition of Mg^2+^ to the cell culture medium. (A) Diagram of pT7 transcription cassettes expressed from plasmids pLl.LtrB-GFP and pGFP, which contain either the Ll.LtrB intron and short flanking exons or just the ligated-exon sequences that would result from Ll.LtrB intron splicing inserted at the same location within the GFP ORF. Red arrows show the location of primers used for RT-PCR and RT-qPCR of Ll.LtrB intron splicing. (B) RNAi knockdown of UPF1. HEK-293 cells were pretreated for 24 h with either scrambled siRNA (black) or UPF1 siRNA (white) to inhibit NMD and then transfected with the pGFP or pLl.LtrB-GFP. At 48 h after transfection of the plasmids, the number of GFP transcripts per cell was measured by RT-qPCR. The immunoblot below the bar graph confirms knockdown of UPF1 protein by the UPF1 siRNA but not by the control scrambled siRNA in HEK-293 cells at 72 h after transfection of the siRNA corresponding to the time at which GFP transcript levels were measured. Equal amounts of cellular proteins were loaded in each lane. (C) RT-PCR assay for RNA splicing of Ll.LtrB transcripts in HEK-293 cells at 24 h after transfection of the indicated plasmids with or without 80 mM Mg^2+^ added to the culture medium. The RT-PCR products were analyzed in a 1% agarose gel, which was stained with ethidium bromide. The positions of the PCR products corresponding to unspliced and spliced pLl.LtrB-GFP RNAs are indicated to the right of the gel. Splicing of the Ll.LtrB intron in HEK-293 cells with 80 mM Mg^2+^ added to the culture medium was confirmed by sequencing across the ligated-exon junction in the PCR product. The experiment was done twice with similar results. (D) GFP fluorescence in HEK-293 cells transfected with plasmids pGFP or pLl.LtrB-GFP + phLtrA with 80 mM MgCl_2_ added to the culture medium. Images were taken 72 h post-transfection. The experiment was done twice with similar results. Abbreviations: E1 and E2, 5’ and 3’ *ltrB* exons, respectively; IRES: internal ribosome entry site; pA, polyadenylation signal; PT7: T7 RNAP promoter.

The finding that Pol II transcripts containing the Ll.LtrB intron are subject to NMD in human cells led us to test whether this barrier could be overcome by using T7 RNAP for Ll.LtrB expression. T7 RNAP transcripts are not capped, polyadenylated, or subject to pre-mRNA processing in the same way as Pol II transcripts and thus are not expected to be subject to NMD [43]. For these experiments, we constructed two T7-promoter-driven GFP expression plasmids, one denoted pGFP-Ll.LtrB containing the Ll.LtrB intron and short flanking exon sequences inserted within the GFP ORF, and the other denoted pGFP containing the ligated-exon sequences that would result from Ll.LtrB intron splicing inserted at the same location (Fig 4A). In both plasmids, the GFP ORF is preceded by an internal ribosome entry site (IRES) to enable GFP expression if the Ll.LtrB intron is spliced. Paralleling the protocol used for BFP-encoding Pol II transcripts in Fig 3, we transfected these GFP-encoding plasmids together with pT7-NLS, which expresses T7 RNAP, into HEK-293 cells that had been pre-treated with the UPF1 siRNA or a scrambled control siRNA, and we measured GFP transcript levels by RT-qPCR at 48 h after transfection of the plasmids. In this case, the T7-GFP ORF control and T7-Ll.LtrB-GFP transcript containing the Ll.LtrB intron were present at similar levels with either the scrambled or UPF1 siRNA, with UPF1 knockdown by the UPF1 siRNA again confirmed by immunobotting (Fig 4B). These findings indicate that a T7-transcript containing the Ll.LtrB intron is not subject to NMD pathway-related degradation in human cells.

The Pol II-transcripts with the Ll.LtrB intron inserted in BFP ORF described in the preceding sections were not spliced in human cells, and this was also the case for the T7 transcripts with the Ll.LtrB intron inserted in the GFP ORF. As we suspected that splicing of the Ll.LtrB-intron might be limited by low Mg^2+^ concentrations in human cells, we tested whether splicing of the T7 transcript containing the Ll.LtrB intron might be induced simply by growing cells in culture medium containing elevated concentrations of MgCl_2_ (Fig 4C). In these experiments, we transfected the three expression plasmids phLtrA, pT7-NLS, and pGFP-Ll.LtrB into HEK-293 cells in culture medium with or without added 80 mM MgCl_2_ and assayed Ll.LtrB intron splicing by RT-PCR of cellular RNAs at 48-h post-transfection. In standard culture medium, the GFP-Ll.LtrB transcript by itself showed no detectable splicing (lane 2), while co-expression of hLtrA led to low levels of splicing (lane 4). Surprisingly, the addition of MgCl_2_ to the culture medium by itself led to a large increase in splicing even in the absence of hLtrA (lane 3). Splicing levels in the presence of both exogenous MgCl_2_ and hLtrA appeared to be somewhat lower than with MgCl_2_ alone (lane 5). Accurate splicing was confirmed by sequencing of the ligated-exon junction in the PCR product. Notably, although the Ll.LtrB intron was spliced under these conditions, we detected no expression of GFP from the spliced transcript, whereas GFP was expressed efficiently from the control transcript containing the ligated-exon junction sequence inserted at the same location in the GFP ORF (Fig 4D). Together, these findings indicate that exogenous MgCl_2_ can by itself induce splicing of the Ll.LtrB intron in human cells, even in the absence of LtrA protein, which is required for Ll.LtrB intron splicing in bacteria [49]. However, T7 transcripts from which the Ll.LtrB intron had been spliced in human cells still appear to be subject to a translational block similar to what was found for RNAP II transcripts in *S*. *cerevisiae* [11].

### The Ll.LtrB intron can retrohome into plasmid and genomic target sites in human cells

**Fig 5:**
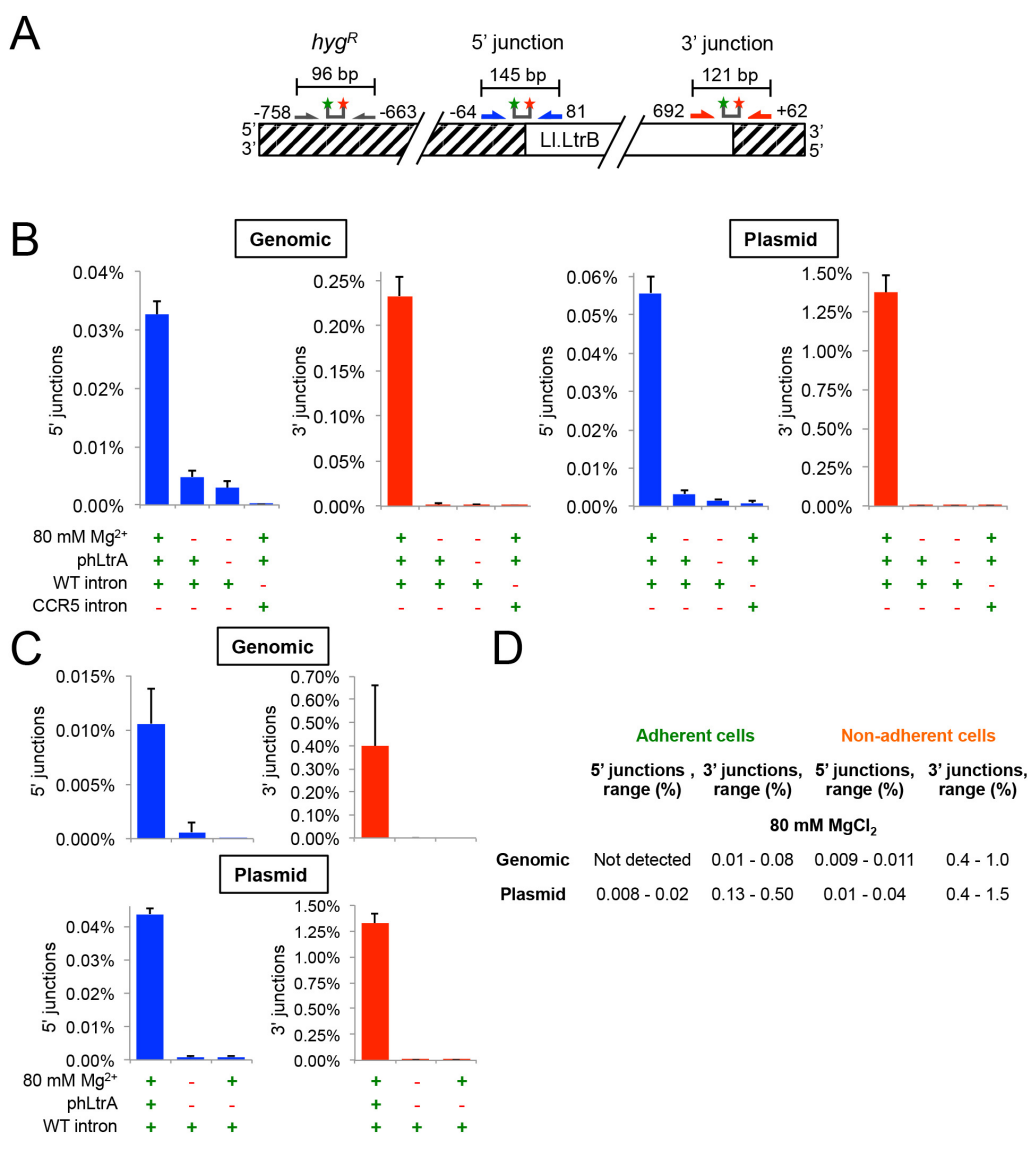
The group II intron Ll.LtrB can retrohome into genomic and plasmid target sites in human cells after addition of Mg^2+^ to the cell culture medium. (A) Diagram of Taqman qPCR assays used to measure retrohoming efficiency. The wild-type Ll.LtrB target site was inserted into the genome of HEK-293 Flp-In cells and cloned in recipient plasmid pFRT for assays of genomic and plasmid retrohoming, respectively. Striped regions indicate DNA from the plasmid used in generating the Flp-In cell line. Arrows and starred bars indicate primers and Taqman probes used for qPCR, respectively. Green and red stars of Taqman probes correspond to fluorophore and quencher moieties, respectively. Frequencies of 5’- and 3’-integration junctions were measured relative to number of copies of the hygromycin-resistance marker (*hyg* ^*R*^) present upstream of the Ll.LtrB target site. (B) and (C) Taqman qPCR assays. HEK-293 Flp-In cells containing the integrated Ll.LtrB target site were transfected with Ll.LtrB expression plasmids plus recipient plasmid pFRT for plasmid assays, as indicated below the bar graphs, and incubated in culture medium supplemented with 80 mM MgCl_2_ for 24 h, prior to recovering total cells (adherent and non-adherent) and isolating total DNA for qPCR assays. Blue and red bars show frequencies of 5’- and 3’-integration junctions (note different scales), respectively, relative to copies of a sequence within the *hyg* ^*R*^ marker. The bar graphs show the average for three experiments with the error bars indicating the SD. (D) Retrohoming frequencies in adherent versus non-adherent cells after 24 h with 80 mM MgCl_2_ added to the culture medium. The values shown are the range of retrohoming frequencies in ≥4 experimental trials based on qPCR assays of 5’- and 3’- integration junctions in genomic or plasmid target sites. Later experiments typically used only adherent cells.

*In vitro*, the LtrA protein can be reconstituted with excised intron lariat RNA to generate RNPs that are active in retrohoming [21]. Thus, we tested whether the excised intron RNA resulting from Ll.LtrB splicing in culture medium containing added MgCl_2_ (80 mM) could be combined with expressed hLtrA to promote retrohoming in human cells. To assess retrohoming in human cells, we used sensitive Taqman qPCR-based assays that quantify both the 5'- and 3'-integration junctions resulting from integration of the Ll.LtrB intron into the wild-type DNA target site (Fig 5A). We tested for retrohoming into a single genomic copy of the wild-type Ll.LtrB homing site in HEK-293 Flp-in cells and in the same cells after co-transfection of a recipient plasmid (pFRT) carrying the same target site. As the transfected plasmid is expected to be present in much higher copy number (~10^4^) [50] than the genomic target site, this protocol enables direct comparison of plasmid and genomic targeting in parallel transfections of the same cells. In these experiments, a 24-h period of polyethylenimine (PEI)-mediated transfection of the expression plasmids was followed by an additional 24-h period in which cells were incubated in growth medium containing 80 mM MgCl_2_. After MgCl_2_ treatment, the cells (both adherent and non-adherent) were collected, and DNA was extracted for qPCR analysis.

Cells receiving all three expression plasmids and 80 mM MgCl_2_ showed significant retrohoming into both genomic and plasmid retrohoming assays (Fig 5B). In three separate experiments, the average retrohoming frequency and standard deviation for the genomic target site measured by qPCR of RNase-treated whole-cell DNA was 0.23 ± 0.02% for the 3'-integration junction and about 7-fold lower, 0.033 ± 0.002%, for the 5'-integration junction [note the different scales of the y-axis for 5’-junctions (blue bars) and 3’ junctions (red bars) in Fig 5B and 5C).] The retrohoming frequencies for cells co-transfected with the recipient plasmid, which is present at ~10^4^ copies per cell and expected to be largely cytosolic [50,51], were substantially higher (1.4 ± 0.1% for the 3’-integration junction and 0.056 ± 0.004%, for the 5' junction). The lower frequency of 5’- than 3’-integration junctions for retrohoming of the Ll.LtrB intron into genomic and plasmid target sites may reflect that a high proportion of the retrohoming events result in the integration of 5’-truncated introns, similar to the situation for human LINE-1 elements where retrotransposition frequently results in 5’ truncations due to abortive reverse transcription [52]. Surprisingly, retrohoming efficiencies with both plasmid and genomic target sites were similar regardless of whether or not the expressed T7 RNAP contained an NLS (S1 Fig). This finding presumably reflects that RNPs resulting from transcription of pLl.LtrB that remains in the cytosol after transfection can still gain access to the genomic target site (S1 Fig; see Discussion).

For retrohoming into the plasmid target site, full-length intron integrations requiring all steps in retrohoming were confirmed by conventional PCR and sequencing of the integration junction in the above experiments (S2 Fig) and more extensively in genetic assays described below. For the genomic target site, the very low frequency of full-length intron integrations (0.033%) made it difficult to recover them from whole-cell DNA by conventional PCR. However, both the 5’- and 3’-integration junctions expected for full-length integrations were detected by Taqman qPCR assays of RNase-treated genomic DNA at levels well above background and with the same excess of 3’ junctions as found in the plasmid assay (Fig 5B and 5C). Additionally, unlike splicing of the Ll.LtrB intron in human cells, which is not dependent upon LtrA protein (see above), retrohoming of the Ll.LtrB intron into both plasmid and genomic DNA target site and the detection of both the 5’- and 3’-DNA integration junctions required the LtrA protein, which is needed for DNA target site recognition as well as reverse transcription (Fig 5B and 5C). Finally, in an important control, no significant retrohoming into the wild-type plasmid or genomic site was detected under any condition for an Ll.LtrB intron retargeted to insert into the *CCR5* gene (Fig 5B). We confirmed that this *CCR5* targetron retrohomes into a plasmid-borne CCR5 target in HEK-293 cells at frequencies of 0.24–0.27% for the 3’-integration junction, but could not detect integrations into the genomic *CCR5* gene.

Although retrohoming frequencies in HEK-293 cells with added Mg^2+^ were relatively high, we observed that the addition of 80 mM MgCl_2_ to the culture medium to promote retrohoming resulted in cellular blebbing, a hallmark of apoptosis [53], with about half of the cells becoming non-adherent and unable to divide in fresh media. Inviable non-adherent cells could potentially have higher targeting rates due to enhanced Mg^2+^ influx due to more permeable cell membranes. Consistent with this possibility, we found that retrohoming frequencies at 80 mM MgCl_2_ were substantially higher in non-adherent cells (3’-integration junctions 0.4–1.0% and 0.4–1.5% for genomic and plasmid target sites, respectively) than in adherent cells (3’-integration junctions 0.01–0.08% and 0.13–0.50% for genomic and plasmid target sites, respectively) (Fig 5D). We tested whether lower MgCl_2_ concentrations, shorter targeting times, or different Mg^2+^ salts could alleviate the deleterious effects of added Mg^2+^, but found that all treatments that improved cell viability decreased retrohoming frequencies to unattractively low levels (S3 Fig). Cell populations in which the Ll.LtrB intron had integrated into the genomic site at 80 mM Mg^2+^ were viable and remained adherent in high MgCl_2_ growth medium indefinitely. Thus, these experiments indicate that the Ll.LtrB intron can retrohome into both plasmid and genomic target sites in viable human cells, the latter at frequencies as high as ~0.1% as measured by 3’-integration junctions, so long as extra Mg^2+^ is added to the culture medium.

### Directed evolution of Ll.LtrB for enhanced retrohoming in human cells

The finding that retrohoming of the Ll.LtrB intron in human cells is limited by low Mg^2+^ concentrations led us to test whether we could select Ll.LtrB intron variants that could retrohome more efficiently at low Mg^2+^ concentrations in human cells. We previously selected Ll.LtrB variants with mutations in the distal stem of domain V (DV) that had 10- to 20-fold higher retrohoming efficiencies in a Mg^2+^-deficient *E*. *coli* mutant, as well as decreased Mg^2+^-dependence for RNA splicing and reverse splicing *in vitro* [36]. However, neither of the two best such variants had increased retrohoming efficiency into genomic or plasmid target sites in HEK-293 cells with or without 80 mM MgCl_2_ added to the culture medium (S4 Fig). We also tested an intron variant that was selected for enhanced retrohoming in *Xenopus laevis* oocyte nuclei, another environment in which low Mg^2+^ concentrations are stringently limiting for retrohoming [10,54]. Although this variant had ~4-fold higher retrohoming efficiency in *X*. *laevis* oocyte nuclei, it did not show higher retrohoming frequencies than wild-type Ll.LtrB in human cells in our assays (S5 Fig). A possible explanation is that these Ll.LtrB variants selected in *E*. *coli* or *X*. *laevis* are optimized for different intracellular environments and Mg^2+^ concentrations than those in human cells. Thus, we attempted to select Ll.LtrB variants with enhanced retrohoming directly in human cells.

**Fig 6:**
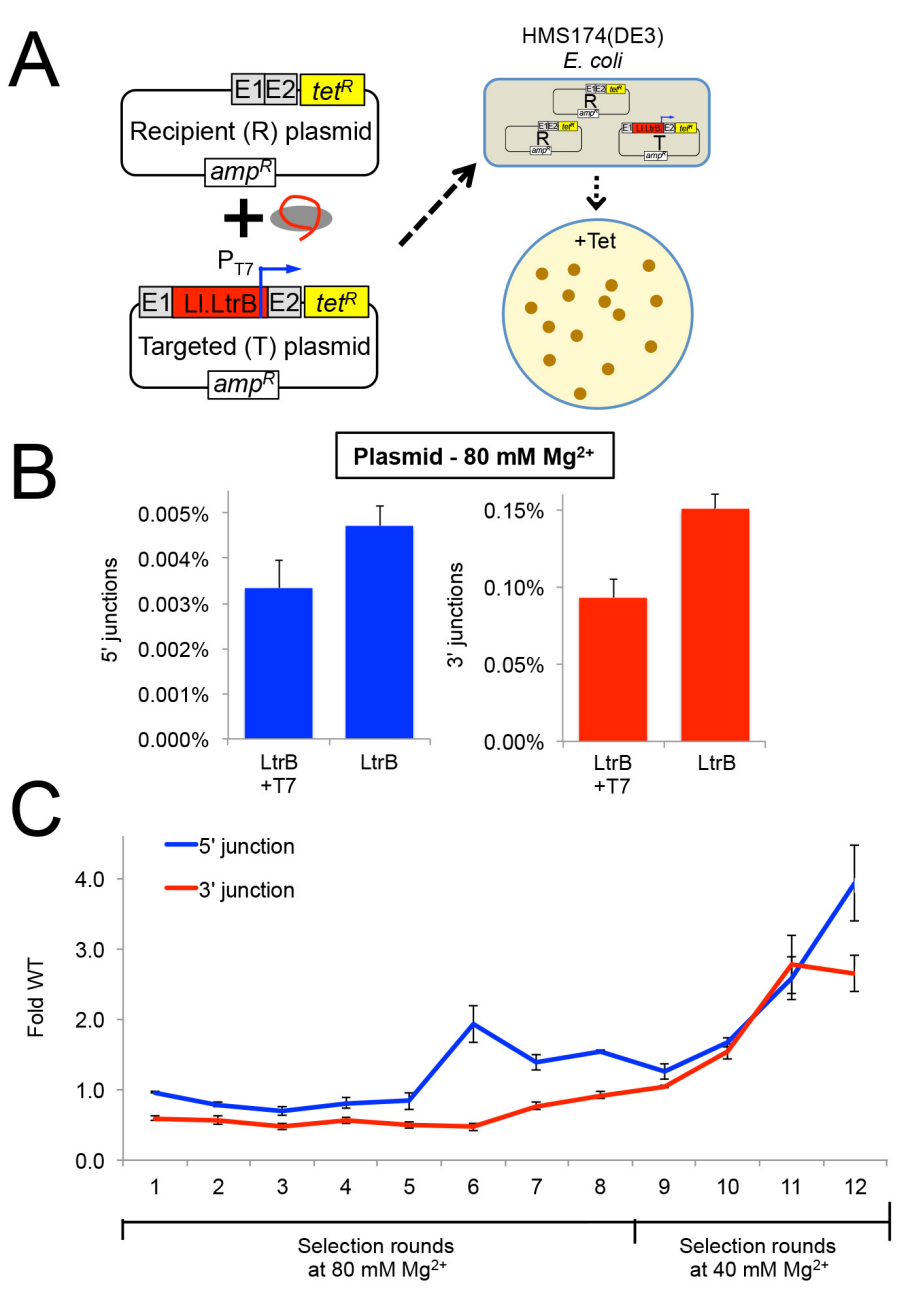
Selection of Ll.LtrB group II intron for retrohoming within HEK-293 cells at different MgCl_2_ concentrations. (A) Diagram of plasmid-based selection for retrohoming in human cells. The three Ll.LtrB expression plasmids, including a derivative of pLl.LtrB in which the expressed intron carries a phage T7 promoter sequence in DIVb, were transfected into HEK-293 cells along with recipient plasmid pBRRQ, which contains the wild-type Ll.LtrB target site cloned upstream of a promoterless *tet* ^R^ gene. After incubating the cells in culture medium supplemented with 80 or 40 mM Mg^2+^ for 24 h, plasmids were isolated and electroporated into *E*. *coli* HMS174(λDE3), which was then plated on LB-agar containing tetracycline. Plasmids were isolated from scraped *E*. *coli* colonies, and introns that had retrohomed into the target site were amplified by PCR using primers that flank the intron and recloned into pLl.LtrB for the next round of selection. (B) Ll.LtrB introns carrying a phage T7 promoter in DIVb have ~70% wild-type retrohoming efficiency in plasmid targeting assays in HEK-293 cells. The bar graphs show retrohoming frequencies assayed by Taqman qPCR of 5’- (blue) or 3’- (red) integration junctions in DNA extracted from adherent HEK-293 cells after 24-h incubation in culture medium supplemented with 80 mM Mg^2+^. Values are the mean for two or three separate transfections on the same day, with the error bars indicating the SEM. (C) The Ll.LtrB intron was evolved for retrohoming into plasmid targets within HEK-293 cells via eight cycles of selection at 80 mM MgCl_2_ with addition of three new mutations per kb between each cycle (rounds 1–8). After round 8, intron variants were selected for an additional four cycles in HEK-293 cells in culture medium supplemented 40 mM MgCl_2_ without mutagenesis (rounds 9–12) to enrich for variants that enhance retrohoming within HEK-293 cells. The retrohoming frequencies for the wild-type Ll.LtrB intron and libraries for rounds 1 to 12 were assayed in parallel by Taqman qPCR for three separate transfections on the same day. The values plotted are the mean with the error bars indicating the SEM.

For directed evolution in human cells, we adapted an *E*. *coli* plasmid-based genetic assay for retrohoming that avoids pitfalls of PCR amplification of low frequency intron-integration events [6]. In this assay, a group II intron carrying a phage T7 promoter retrohomes into a target site cloned on a recipient plasmid upstream of a promoterless tetracycline-resistance gene (*tet* ^*R*^) resulting in a Tet^R^ plasmid that can be selected by transformation of human cell DNA preparations into *E*. *coli* (Fig 6A). The retrohoming efficiency of the Ll.LtrB variant containing the phage T7 promoter in HEK-293 cells supplemented with 80 mM MgCl_2_ was ~70% that of the wild-type intron as measured by Taqman qPCR in plasmid targeting assays (Fig 6B). For *in vivo* selections, the three Ll.LtrB intron expression plasmids were co-transfected with the recipient plasmid into HEK-293 cells, which were then incubated in culture medium with 80 mM MgCl_2_. After 24 h, plasmids were extracted from the HEK-293 cells by an alkaline-lysis procedure and electroporated into *E*. *coli* HMS174(λDE3) to select for Tet^R^ colonies, which were screened by colony PCR and sequencing of both 5’- and 3’-integration junctions to confirm retrohoming of the full-length Ll.LtrB intron into the DNA target site (S6 Fig). In controls, no retrohoming was detected by this assay in HEK-293 cells transfected with the same plasmids, but incubated in culture medium without 80 mM MgCl_2_. This control confirms that retrohoming detected in the assay occurred in human cells and not after transformation of the donor and recipient plasmids into *E*. *coli*, and it provides further evidence that addition of MgCl_2_ to the culture medium is needed to stimulate Ll.LtrB retrohoming in human cells.

We used the HEK-293 cell plasmid selection system to perform eight rounds of *in vivo* directed evolution in culture medium supplemented with 80 mM MgCl_2_ via an adaptive walk in which introns that retrohomed into the plasmid target site in each round were amplified by PCR at a relatively high mutagenesis frequency of 3 mutations per intron per round prior to re-cloning into the expression vector for the next round (Fig 6C). After eight rounds, we increased the stringency of the selection by reducing the MgCl_2_ concentration to 40 mM and performed four additional selection cycles without the addition of new mutations between cycles (rounds 9–12). The retrohoming efficiency of the selected pools relative to that of the wild-type intron assayed in parallel increased slowly from rounds 6 to 9 and somewhat more rapidly during rounds 10 to 12. After the 12 rounds of selection shown in the Figure, an additional three rounds of selection with and without mutagenesis gave no further improvement in retrohoming efficiency of the pools relative to the wild-type intron at 40 mM MgCl_2_. As described below, high-throughput sequencing indicated that this plateau in retrohoming efficiency reflected that a small number of mutations that moderately enhance retrohoming had overtaken the pool at round 12 and could not be substantially improved by other mutations that were positively selected at either 40 or 80 mM Mg^2+^.

### High-throughput sequencing of Ll.LtrB introns evolved in HEK-293 cells

Although the mutant pools were not increasing in activity at a rapid pace, the possibility remained that individual mutations or combinations of mutations in the pool had enhanced retrohoming. To investigate the mutational diversity of the evolution cycles, we used Pacific Biosciences single-molecule sequencing (PacBio RS), which provides long read lengths (1,000–15,000 nt), combined with circular consensus sequencing (CCS), which compensates for sequencing errors by using rolling-circle amplification to generate concatameric-sequencing reads of the same molecule [55]. An advantage of PacBio RS is that it reads single-molecules directly and thus alleviates problems stemming from formation of molecular hybrids during PCR, which can over-estimate the number of unique sequences in molecular diversity experiments [56,57]. We further avoided formation of PCR hybrids by preparing the sequencing libraries directly from Tet^R^-positive recipient plasmids that contained integrated introns without PCR.

**Fig 7:**
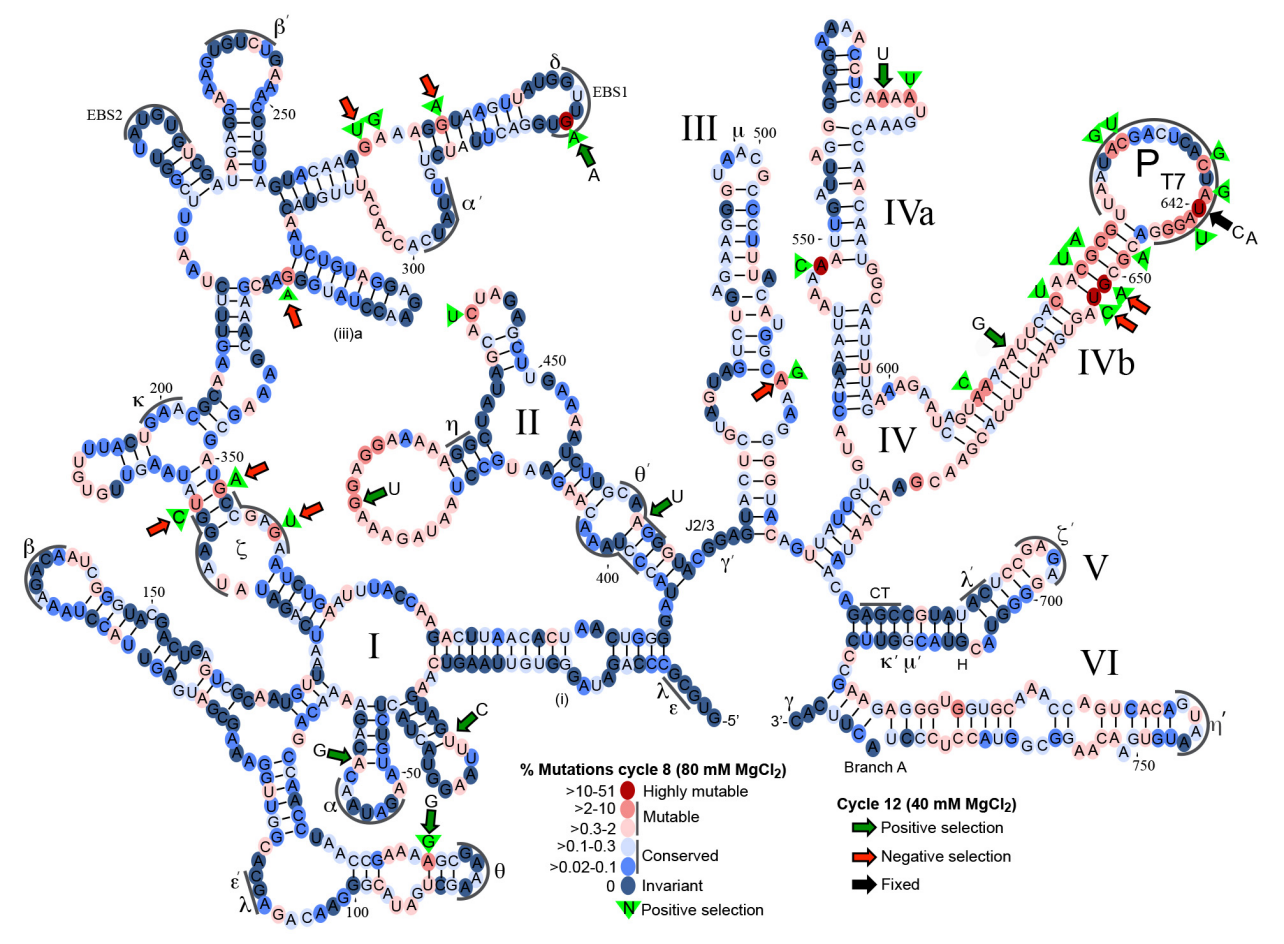
Mutational fitness map of the Ll.LtrB intron during directed evolution in human cells. Secondary structure diagram of the Ll.LtrB intron showing mutation frequencies based on Pacific Biosciences RS circular consensus sequencing after 8 and 12 cycles of directed evolution in HEK-293 cells in culture medium supplemented with 80 mM or 40 mM MgCl_2_. Round 8 was assessed with 1,395 full-length reads, with the results presented as a color-coded heat map on a secondary structure diagram of the Ll.LtrB intron. Dark to light blue ovals represent conserved nucleotide positions with mutations present in 0–0.3% of the population. Pink to red ovals indicate mutable nucleotide positions with mutations present in >0.3–60% of the population. Green triangles with nucleotide inscribed, indicate positive selection in rounds 1–8 with the indicated nucleotide comprising >80% of the mutations at that position. Round 12 was assessed with 3,069 full-length reads with large colored arrows indicating positions at which the indicated mutation increased (green arrows) or decreased (red arrows) in frequency by >2-fold in round 12 after selection at 40 mM Mg^2+^ relative to round 8 after selection at 80 mM Mg^2+^. The black arrow at position 642 indicates that the mutations shown at this position were fixed in 99% of the population in round 12. Greek letters with sequence delineated below indicate motifs involved in long-range tertiary structure interactions.

We first sequenced retrohomed introns from round 8 (NCBI SRA database, accession number SAMN03342363) and generated a fitness map that displays the degree of conservation of each nucleotide as a heat map on a secondary structure diagram of the Ll.LtrB intron (Fig 7). The degree of conservation of different nucleotides displayed a wide range and is shown with a scale ranging from dark to light blue for conserved sites (0–0.3% mutations) and from pink to red for mutable sites (>0.3–51% mutations) (Fig 7). On average, the round 8 mutant pool contained 4.4 mutations per intron. The majority of nucleotides (551 of 776) in the intron were conserved (dark or light blue) over eight cycles of directed evolution. Regions required for ribozyme activity (*e*.*g*., the catalytic triad in DV, J2/3, which interacts with DV to form the active site, the branch-point A residue in DVI, and the 5’ and 3’ ends of the intron) were invariant, with the exception of a few nucleotides previously shown to be less constrained within those regions (*e*.*g*., the dinucleotide bulge in DV). The most variable regions were DIVb, which lies outside the catalytic core, and the two terminal loops of DII. DIVa, which contains a high-affinity LtrA-binding site, showed strong conservation of most nucleotides found to be critical for LtrA binding (positions 557, 559, 561–564), but not position 556 [58,59]. A mutation at position 548 in an internal loop in DIVa was positively selected (green triangle) and could affect LtrA binding.

Although many of the nucleotide changes after 8 cycles of selection appear to be neutral, as they do not bias towards any specific nucleotide, mutations at 25 sites were positively selected (nucleotides within green triangles in Fig 7), meaning that >2% of the population had a mutation at that position of which >80% had the indicated base. Two of the positively selected mutations were within sequence elements involved in long-range tertiary interactions within the catalytic core (ζ and θ’), while six of the positively selected mutations disrupted or weakened base-pairing interactions. Mutations at two sites became highly prevalent in the population (>27%). The first was a G282A mutation in EBS1, which changes a UG to a UA base pair at position -4 of the EBS1/IBS1 interaction between the intron and 5’ exon and had been shown previously to result in an ~50% increase in the efficiency of reverse splicing into a DNA target site *in vitro* [60]. The second was intron position 642, which was mutated in 51% of the population at round 8 and 99% at round 12 (black arrow). At round 8, 63% of the mutations at position 642 were U to A and the other 37% were U to C. Position 642 is located two nucleotides upstream of the transcription start site of the T7 promoter inserted for selection purposes within DIVb. Although mutations at this position could in principle simply attenuate the T7 promoter [61], leading to less T7-induced toxicity in our *E*. *coli* assay, experiments below show that the selected mutations increase retrohoming efficiency in human cells in Taqman qPCR assays. The T7 promoter "TATA-box" region has been shown to interact with TFIID and Pol II in HeLa cell extracts [62,63], and mutations in this canonical “TATA box” could potentially decrease TFIID- and Pol II-binding, leading to increased production of full-length intron transcripts, or could affect retrohoming by some other mechanism. Finally, while the distal stem of DV was mutable, as previously shown in *E*. *coli* selections [36], it was not the site of mutations undergoing positive selection for retrohoming in human cells. This finding is in agreement with the results of S4 and S5 Figs, which show that mutations in the distal stem of DV that increased retrohoming efficiency in *E*. *coli* or *X*. *laevis* oocyte nuclei, did not increase retrohoming frequency in human cells.

To determine whether the mutations that were positively selected in HEK-293 cells at 80 mM Mg^2+^ (rounds 1–8) were enriched further after more stringent selection without mutagenesis at 40 mM Mg^2+^ (rounds 9–12 (Fig 6C), we sequenced retrohoming products from round 12 (NCBI SRA database, accession number SAMN03342364). In Fig 7, positions at which the mutation frequency increased or decreased by >2-fold from cycle 8 at 80 mM Mg^2+^ to cycle 12 at 40 mM Mg^2+^ are indicted by large green or red arrows, respectively. Surprisingly, over half (9 of 16) of the positively selected nucleotides that comprised >5% of the population in cycle 8 decreased to less than 0.3% of the population in cycle 12 (red arrows). Conversely, six of the eight positively selected mutations that comprised >4% of the population in cycle 12 (green arrows with indicated nucleotide) were not prevalent in the population at cycle 8 (<2%). Four of the eight mutations that were positively selected in round 12 weakened or disrupted base pairs in the intron secondary structure. Two positions that were under positive selection at both 80 and 40 mM Mg^2+^, the EBS1 mutation G282A and the DIVb mutations U642C and U642A, were present in 64 and 99% of the population, respectively, in cycle 12.

**Fig 8:**
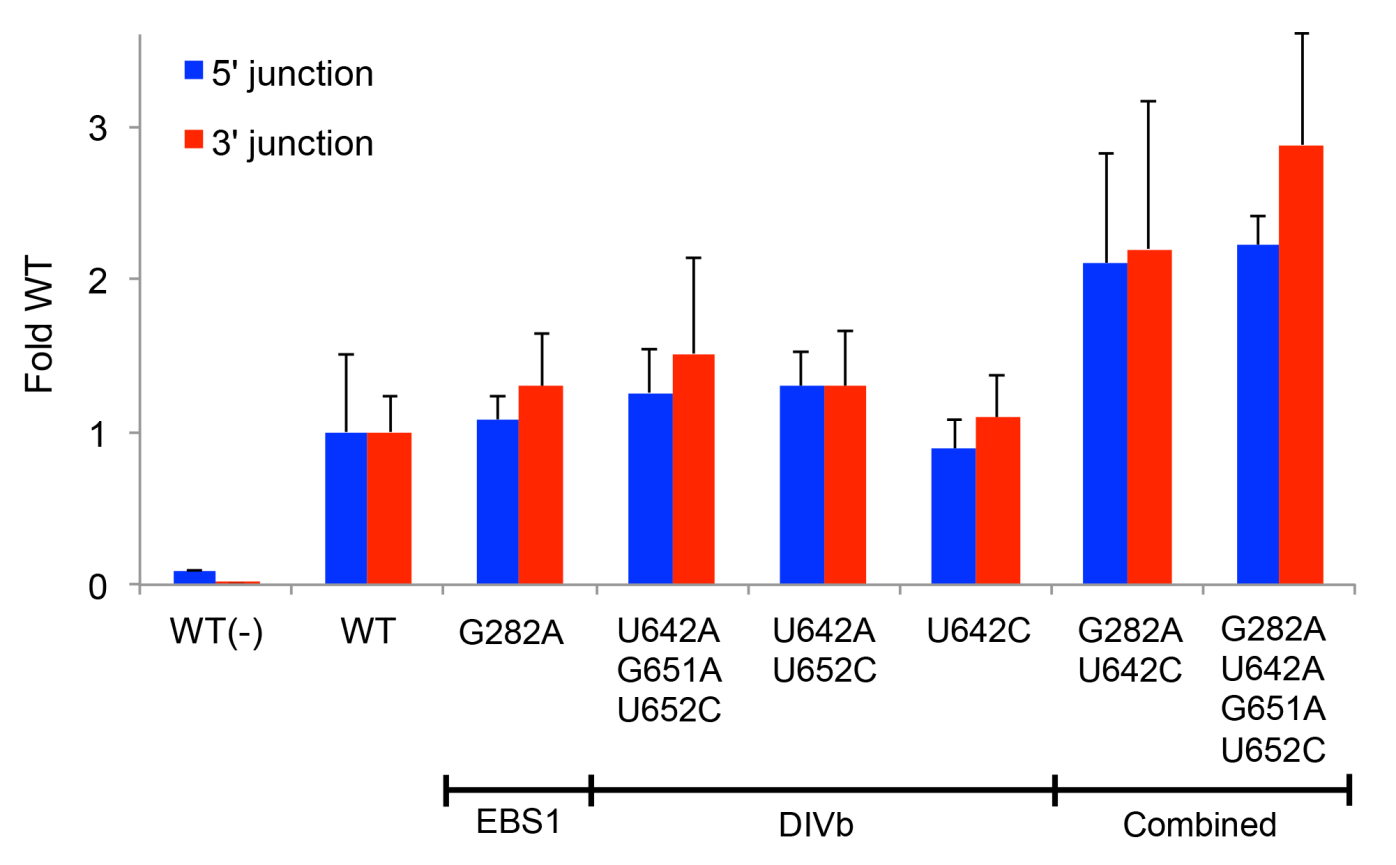
Retrohoming frequencies of Ll.LtrB variants containing positively selected mutations identified by PacBio sequencing. The prominent mutations in EBS1 (G282A) and DIVb (position 642) were combined with other positively selected mutations that showed covariation and tested for retrohoming into a plasmid target site in HEK-293 cells in culture medium supplemented with 80 mM MgCl_2_. Retrohoming frequencies were measured at 24 h after transfection of the expression plasmids in adherent HEK-293 cells by Taqman qPCR assays of the 5’- and 3’-integration junctions relative to the *hyg* ^*R*^ marker adjacent to the target site. The bar graphs show retrohoming frequencies of the variants relative to that of the wild-type intron. Values with error bars are the mean ± SD for at least three experimental trials. Values without error bars were tested once. The negative control WT(-) is the wild-type intron tested without additional MgCl_2_ in the culture medium. Retrohoming frequencies for the wild-type intron measured by Taqman qPCR of 3’-integration junctions ranged from 0.06–0.11% in this series of experiments.

Finally, we identified the top sequencing reads present at highest frequency in cycles 8 and 12 (S1 Table). Many of these contained similar mutations that are candidates for increasing retrohoming activity in human cells. Combinations of these prevalent mutations were tested for linkage disequilibrium (S2 Table) to assess covariation between mutations. The majority of mutation pairs had *D'* values close to 0, indicating equilibrium, but three mutations in DIVb (U642A, G651A, and U652C) compared in pairwise combinations had *D'* values between 1 and 2.3, suggesting strong covariation. A number of Ll.LtrB variants that were most prevalent in the population and/or contained positively selected nucleotides were assayed for retrohoming in HEK-293 cells with 80 mM MgCl_2_ added to the culture medium. Ll.LtrB variants having only the mutations G282A (EBS1) or any of the DIVb mutations (U642A, G651A, U652C) had retrohoming efficiencies similar to or no greater than 50% better than wild type (Fig 8). However, the combinations of G282A (EBS1) and either U642C or U642A-G651A-U652C in DIVb had two- to three-fold higher retrohoming frequencies than the wild-type intron (Fig 8). These findings confirm that selections yielded beneficial mutations that increase retrohoming efficiency with added Mg^2+^ in human cells. However, all of the beneficial mutations identified lie outside the group II intron catalytic core, the most critical positions of which were invariant in the human cell selections.

### Synthetic shuffling of mutations leads to enhanced retrohoming in human cells

**Fig 9:**
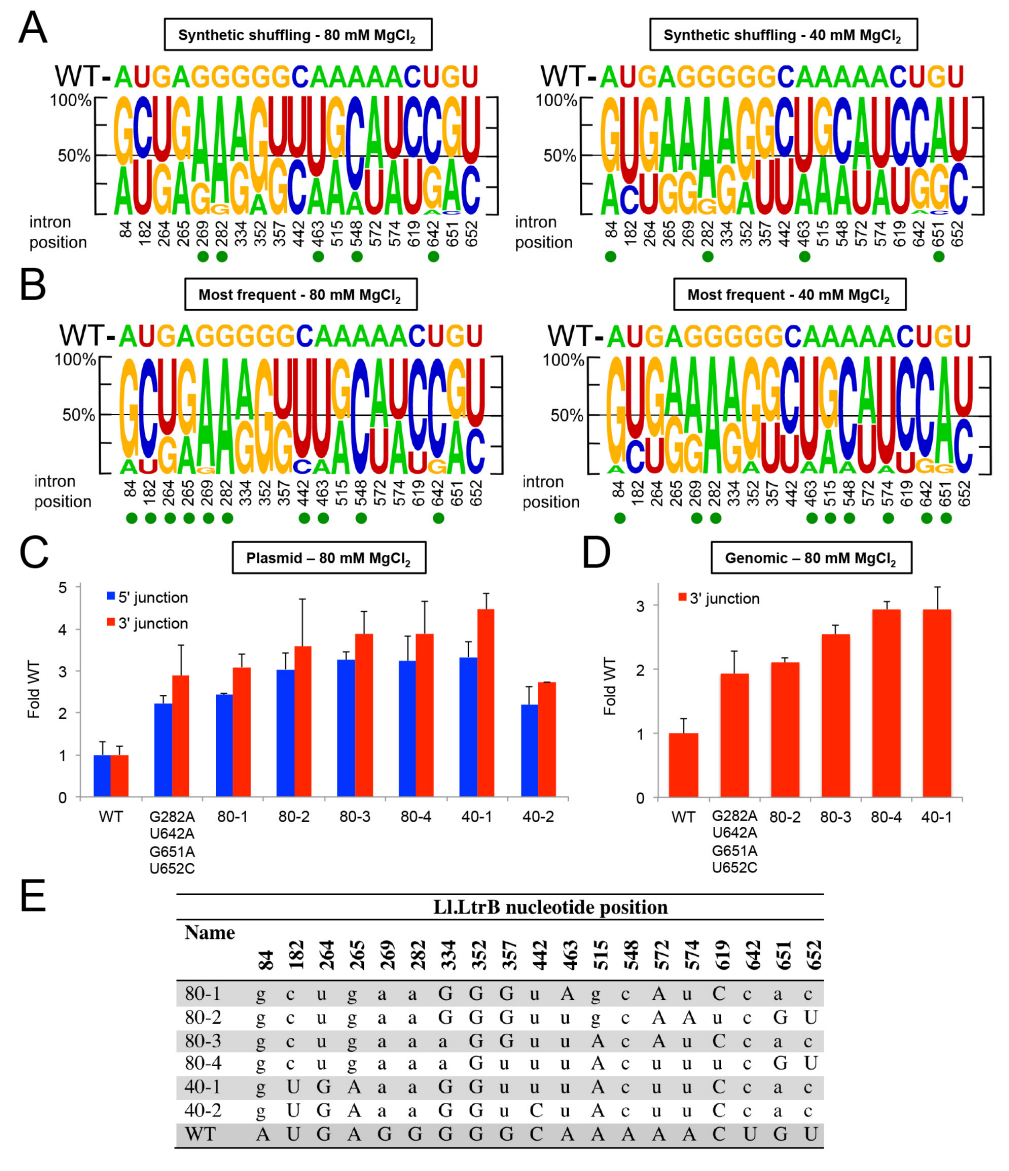
Synthetic shuffling of positively selected mutations identifies Ll.LtrB variants with enhanced retrohoming into plasmid and chromosomal target sites in human cells. Optimal combinations of mutations were identified by the recombination-based technique of synthetic shuffling. A synthetic shuffling library of Ll.LtrB variants was constructed in which mutations at 18 positions that were under positive selection (>80% of one nucleotide type in >5% of the population in selection rounds 8 or 12 (subsets of the nucleotides indicated by green triangles or green or black arrows in Fig 7) were doped fifty percent against the wild-type nucleotide and position 642 was randomized. Sanger sequencing showed that the starting frequency of each nucleotide in the initial library was as expected for the degree of doping or randomization. The introns were selected in HEK-293 cells for four cycles of retrohoming (S7 Fig) with either 80 or 40 mM MgCl_2_ added to the culture medium. Pacific Biosciences RS circular consensus sequencing was used to identify the mutations present in the populations with 4,770 and 4,768 reads obtained for the selections at 80 and 40 mM MgCl_2_, respectively. (A) Sequence logos depicting nucleotide percentages in the population at the indicated positions after four rounds of selection at 80 and 40 mM Mg^2+^, and (B) sequence logos depicting the nucleotide percentages in variants that appear >3 times in the PacBio sequencing in the same selections. The size of the nucleotide indicates its frequency in the population. The wild-type (WT) nucleotide is indicated above the logo, and nucleotide position number is indicated beneath the logo. A green dot indicates that the mutant nucleotide increased in frequency to at least 60% of the population. (C) and (D) Retrohoming frequencies measured by Taqman qPCR for variants identified by synthetic shuffling. The bar graphs in (C) show retrohoming frequencies for four variants from the 80 mM MgCl_2_ selection and two from the 40 mM MgCl_2_ (sequences shown below) into a plasmid target site in HEK-293 cells with 80 mM MgCl_2_ added to the culture medium. The wild-type intron and the best variant from Fig 8, which contained only the prevalent EBS1 and DIVb mutations (G282A, U642A, G651A, U652C), were assayed in parallel. The bar graphs in (D) show retrohoming frequencies into the genomic HEK-293 cell target site with 80 mM MgCl_2_ added to the culture medium for the best four variants from panel (C) compared to wild-type and the best variant from Fig 8. The values are the average for three experiments, with the error bars indicating the SD. The retrohoming frequency of the wild-type intron ranged from 0.034–0.050% in the assays of panel (C) and from 0.017–0.026% in the assays of panel (D). (E) The most prevalent variants identified by PacBio sequencing from synthetic shuffling after four rounds of selection in HEK-293 cells with 80 and 40 mM MgCl_2_ added to the culture medium. The nucleotide position in the Ll.LtrB intron is indicated at the top, and the table shows the nucleotide sequence at that position in the variants. Upper case letters indicate the wild-type nucleotide, and lower case letters indicate mutant nucleotides.

While the PacBio deep sequencing identified some combinations of Ll.LtrB mutations that increase retrohoming frequency in human cells, separately testing every conceivable combination of mutations is an inefficient means of identifying the best variants for human cells. Instead, we turned to synthetic shuffling [64] of high frequency mutations identified from the fitness maps to screen many mutation combinations at once. Based on the sequencing of variants from rounds 8 and 12 (Fig 7), we generated a rationally designed synthetic shuffling mutagenesis library by assembly PCR [65]. The library was constructed to test combinations of mutations that showed positive selection and high penetrance during the initial directed evolution (>80% one nucleotide type present in >5% of the population; subsets of the nucleotides indicated by green triangles or green or black arrows in Fig 7). The library consisted of Ll.LtrB introns in which eighteen such positively selected nucleotides were doped at a 1:1 ratio of the selected to the wild-type nucleotide and position 642 in DIVb was randomized. The library was selected for four cycles of retrohoming in HEK-293 cells at either 80 or 40 mM MgCl_2_ and tested for retrohoming efficiency compared to the wild-type intron at both Mg^2+^-concentrations after each cycle. Both selections gave pools of Ll.LtrB variants with increased activity relative to the wild-type intron (S7 Fig), and we then performed PacBio sequencing of the fourth cycle pool for each of the selections (NCBI SRA database, accession numbers SAMN03342365 and SAMN03342366). The sequencing showed that specific mutations were selected at a number of positions, but these positively selected mutations differed for the selections done at the two different Mg^2+^-concentrations (Fig 9A).

To identify those variations associated with the highest retrohoming activity, we generated separate sequence logos for variants that appeared at least three times in the deep sequencing (Fig 9B). While the positions that were shifting towards the mutant nucleotide were shared between the total sequence reads versus just the highest prevalence sequence reads, the shifts were more pronounced in the latter. Both the EBS1 position 289 and DIVb position 642 mutations were present in 100% of the highest frequency variants. We assayed a number of these high prevalence variants for retrohoming in HEK-293 cells (Fig 9C–9E). All of the variants had 3–4 fold higher frequencies for retrohoming into the plasmid target site than did the wild-type intron. When we tested the best of these variants for retrohoming into the genomic target site, we found that variants 80–4 and 40–1 had about three-fold increased retrohoming frequencies. Although these variants were the best we found, they were only marginally better than the EBS1/DIVb mutation combinations tested in Fig 8. These findings suggest that the additional positively selected mutations outside EBS1 or DIVb contribute small fitness effects that together lead to increased retrohoming frequencies. The small contributions to enhanced retrohoming by these mutations is consistent with their relatively slow accumulation during the selections compared to the driving mutations in EBS1 and DIVb.

## Discussion

Here we show that a mobile group II intron, the *L*. *lactis* Ll.LtrB intron, can retrohome into a chromosomal DNA site in human cells. To do so, we developed a mobile group II intron expression system that overcomes barriers to group II intron proliferation in eukaryotic nuclear genomes, including suboptimal codon usage and translational repression of the intron-encoded RT, NMD of group II intron-containing RNAs, and suboptimal Mg^2+^ concentrations. NMD was overcome by using phage T7 RNAP rather than Pol II to express the group II intron RNA, while suboptimal codon usage and translational repression were overcome by separately expressing a human codon-optimized group II intron RT from a separate Pol II-transcript. The remaining barrier, suboptimal intracellular Mg^2+^ concentrations in eukaryotic cells, was overcome simply by adding 80 mM MgCl_2_ to the cell culture medium. Retrohoming in human cells was demonstrated by sensitive Taqman qPCR assays of both the 5’- and 3’-integration junctions for both plasmid and chromosomal DNA target sites and by conventional PCR and sequencing of recipient plasmids containing fully integrated intron with both of the expected integration junctions. The expression system workarounds enabled the Ll.LtrB intron to splice and retrohome into both plasmid and chromosomal target sites in viable human cells at frequencies up to ~0.5% and ~0.1%, respectively. However, *in vivo* selections and synthetic shuffling of positively selected mutations gave only modest further improvements in retrohoming efficiency that still required added Mg^2+^ in the cell culture medium. The latter findings suggest that low Mg^2+^ concentrations constitute an effective natural barrier to group II intron proliferation in human cells that is not readily overcome by selecting group II intron variants and may be a major factor in why mobile group II introns failed to persist as such in eukaryotic nuclear genes.

The finding that Pol II transcripts containing the Ll.LtrB intron are selectively degraded by NMD in human cells (Fig 3) extends previous findings for *S*. *cerevisiae* and suggests that this defense mechanism against mobile group II introns is used generally in eukaryotes [11]. The Ll.LtrB-intron contains multiple stop codons in all three reading frames and could be degraded either by the exon-junction complex (EJC)-dependent NMD pathway, if the Ll.LtrB-containing transcript contains cryptic spliceosomal splice sites, or by non-EJC-dependent NMD mechanisms, which are known to operate in mammalian cells [66]. By contrast, a T7 RNAP transcript containing the intron is not subject to NMD and accumulates to the same levels as a parallel control transcript lacking the intron (Fig 4). Although the T7 RNAP-synthesized Ll.LtrB transcript accumulates to levels sufficient to support retrohoming in human cells, it has a 5’-triphosphate and up-regulates interferon-response genes, such as RIG-I and IFIT1, which may lead to its sequestration or degradation [45]. Suppression of these innate immune responses could lead to higher levels of T7 RNAP transcripts and retrohoming in human cells than observed here.

The finding that supplementation of the cell culture medium with 80 mM Mg^2+^ was by itself sufficient to enable splicing and retrohoming of T7 transcripts containing the Ll.LtrB intron indicates that intracellular Mg^2+^ concentrations are limiting for these processes in human cells [67]. This finding extends previous work showing that group II intron RNPs microinjected into *Xenopus laevis* oocyte nuclei and *Drosophila* and zebrafish embryos could retrohome efficiently into plasmid target sites only when Mg^2+^ was injected in addition to the group II intron RNPs [10]. In contrast to yeast, where transcripts containing the Ll.LtrB group II intron RNA are spliced but not translated [11,35], we observed no detectable splicing of Ll.LtrB-transcripts in human cells without Mg^2+^ supplementation, even when intron RNA degradation by NMD was suppressed. The *Pylaiella littoralis* Pl.LSU/2 group II intron could also splice in yeast but not in a human cell line (HCT116 cells; [12]). Thus, the intracellular environment in human cells under normal growth conditions appears to be less amenable to group II intron splicing than it is in yeast.

Surprisingly, the Mg^2+^-stimulated splicing of the Ll.LtrB intron in human cells neither required nor was enhanced by the LtrA protein, which is needed for group II intron splicing in bacteria or *in vitro* [21,49]. This IEP-independent splicing could reflect either self-splicing of the Ll.LtrB intron or that human cellular proteins can replace LtrA to stabilize the active intron RNA structure. An intriguing possibility is that the Ll.LtrB intron can be spliced in human cells by a protein evolutionary related to LtrA, such as a LINE-1 or telomerase RT, or the spliceosomal protein Prp8, which evolved from a group II intron-like RT [32].

Although dispensable for splicing in human cells, the group II intron RT remains essential for retrohoming, where it contributes to DNA target-recognition and is required for target DNA-primed reverse transcription [22,68]. The expressed LtrA protein could in principle bind to the group II intron RNA either before or after splicing, the latter being analogous to the reconstitution of active group II intron RNPs *in vitro* by binding of purified LtrA to self-spliced intron lariat RNA [21]. The similar retrohoming efficiencies when T7 Pol was expressed with or without an NLS (S1 Fig) indicate that nuclear transcription and splicing of Ll.LtrB RNA to produce functional RNPs is not required for retrohoming and can also occur from transfected plasmids that remain in the cytosol. Free Mg^2+^ concentrations may be higher in the cytoplasm than the nucleus, where Mg^2+^ is sequestered by chelation to chromosomal DNA [69], thereby favoring group II intron RNA splicing and RNP assembly in that compartment rather than the nucleus. If so, group II intron RNPs may gain access to chromosomal DNA either passively during mitosis or by using a pre-existing RNP transport system. Both mechanisms have been suggested for LINE-1 and other non-LTR-retrotransposon RNPs, which are assembled in the cytoplasm but must gain access to the nucleus for retrotransposition [70–72].

Unlike retrohoming of the Ll.LtrB intron in bacteria, we found that retrohoming of the Ll.LtrB intron into both genomic and plasmid target sites in human cells yields an excess of 3’- over 5’-integration junctions detected by Taqman qPCR assays (7–49 fold; Figs 5B–5D and S1). This excess of 3’-integration junctions could reflect the integration of 5’-truncated introns similar to human LINE-1 elements, whose retrotransposition frequently results in the integration of 5’-truncated elements due to abortive reverse transcription [52]. For both group II introns and LINEs, a high frequency of 5’ truncations during retrotransposition could reflect a combination of barriers to reverse transcription, such as RNA-binding proteins, RNase cleavage of the intron or LINE RNA during or prior to cDNA synthesis, and the ability to ligate truncated cDNAs to upstream chromosomal DNA by non-homologous end-joining (NHEJ) mechanisms, which are not active in *E*. *coli* [73–75]. The excess of 3’-integration junctions for the Ll.LtrB intron could also reflect retrohoming of excised linear intron RNAs, which can carry out only the first step of reverse splicing, resulting in the attachment of the 3’ end of the intron RNA to the 3’ exon; TPRT would then yield a cDNA copy of all or part of the linear intron RNA that is ligated to the 5’ exon by NHEJ but could also potentially remain unattached [73,74]. Linear intron RNAs may be generated either by hydrolytic splicing induced by Mg^2+^ supplementation in the absence of LtrA protein or by debranching of lariat RNAs, possibly via the same enzyme (Dbr1) that functions in the debranching and turnover of spliceosomal intron lariats [76]. The latter could be yet another eukaryotic defense against the proliferation of mobile group II introns.

The newly developed mobile group II intron expression system enabled us to select directly for Ll.LtrB intron variants that could retrohome more efficiently in human cells. To do so, we used a plasmid-based mobility assay that enabled selection for low frequency retrohoming events via *E*. *coli* transformation and combined it with the long reads of the PacBio RS circular consensus sequencing to identify mutations under positive selection in the evolving populations. Selections at 80 and 40 mM Mg^2+^ showed that the majority of intron nucleotides were conserved and nucleotides that form the intron RNA’s active site were highly conserved or invariant. Variations were found mainly in terminal loops and at a few scattered positions within the intron. Two mutations, one strengthening the EBS1/IBS1 interaction between the intron and 5’ exon, and the other near the T7 promoter sequence inserted in DIVb, saturated the pool but gave only ~2-fold higher retrohoming efficiency, and other positively selected mutations did not confer substantial additional benefit, even in synthetic shuffling experiments to select for optimal combinations of mutations. Further, mutations selected at 80 mM Mg^2+^ differed from those selected at 40 mM Mg^2^, and Ll.LtrB intron variants selected for enhanced retrohoming in Mg^2+^-deficient *E*. *coli* [36] or *X*. *laevis* oocyte nuclei [54] did not show increased retrohoming frequencies in HEK-293 cells. The latter findings may reflect competing effects of altering Mg^2+^-binding at different sites on intron RNA folding, so that variants selected at one low Mg^2+^ concentration are not well suited to function at other low Mg^2+^ concentrations. Previous studies in which variants of the *Azoarcus* group I intron ribozyme were selected under different conditions showed that different combinations of mutations confer fitness for different environments [77,78].

It is possible that very rare mutations not sampled in our selections, different selections, selections with another group II intron, or rational redesign of the group II intron catalytic core based on X-ray crystal structures could yield group II intron variants that retrohome at high frequencies in eukaryotic cells. Until such time, our findings for the Ll.LtrB intron suggest that barriers to group II intron retrohoming in human cells are not readily overcome by mutational variation and selection, possibly reflecting that the group II intron catalytic core cannot be modified readily to function efficiently at lower Mg^2+^ concentrations. The latter could explain why group II introns failed to evolve into a form that could function in eukaryotes without fragmentation into spliceosomal introns and the spliceosome.

Although the Ll.LtrB intron works very efficiently for gene targeting in bacteria [9], its targeting efficiency via retrohoming in human cells is substantially lower than those for current methods using CRISPR/Cas9, zinc-finger nucleases or TALEN-based systems [79]. Additionally, retrohoming of the Ll.LtrB intron in human cells requires the addition of Mg^2+^ to the culture medium, which stresses the cells. Nevertheless, gene targeting efficiencies for the Ll.LtrB intron of near 0.1% might be sufficient for gene targeting applications and could potentially be increased substantially by stable rather than transient expression of the group II intron expression plasmids and/or by suppression of innate immune responses and lariat debranching enzyme. It also remains possible that other group II introns can be found that function more efficiently in human cells than does Ll.LtrB. Finally, as DNA target site recognition by mobile group II introns is not dependent upon ribozyme activity, the ability of group II intron RNPs to recognize a DNA target site in the human genome at appreciable frequency as found here suggests they could be used analogously to CRISPR/Cas9 nuclease-null mutants to localize group II intron RT fusion proteins or modified group II intron RNAs with different functionalities to desired chromosomal locations [80].

Mobile group II introns are thought to have evolved in bacteria where the intracellular Mg^2+^ concentrations are higher than in eukaryotes [1,36,81,82]. They are hypothesized to have entered an ancestral pre-eukaryote, likely an archaeon, with eubacterial endosymbionts that gave rise to mitochondria and chloroplasts, invaded the nucleus, proliferated as mobile elements, and then degenerated with group II intron domains evolving into snRNAs that reconstitute to form the catalytic core of the spliceosome [4,34]. Based on their discovery that Pol II transcripts containing the Ll.LtrB group II intron are subject to NMD and translational repression, Belfort and coworkers hypothesized that translational repression resulting from group II intron insertion into protein-coding genes contributed to group II intron loss from eukaryotic nuclear genomes and their evolution into spliceosomal introns [11,35].

Considered in the context of the above hypotheses, our results suggest that the ancestral eukaryote must have had relatively high intracellular Mg^2+^ concentrations that could support proliferation of group II introns in protein-coding genes by retrohoming and that lowering of intracellular Mg^2+^ concentration in eukaryotes may have been an evolutionary response to selective pressure to restrict group II intron proliferation. Mammals use an analogous defense mechanism based on iron limitation as part of an innate immune response to bacterial infections [83]. In this scenario, a decrease in intracellular Mg^2+^ concentrations in ancestral eukaryotes would have strongly inhibited group II intron splicing, thereby increasing selective pressure against retaining group II introns as such in protein-coding genes. The evolution of the nuclear membrane, itself hypothesized to be an evolutionary response to group II intron invasion [3], had the additional advantage of sequestering group II introns into a separate compartment where free Mg^2+^ concentrations are further decreased by chelation to DNA and chromatin, while enabling the cytosol to maintain higher Mg^2+^ concentrations for other cellular processes [36,67]. A lower free Mg^2+^ concentration in the eukaryotic nucleus would confer immunity from group II introns that are sporadically acquired by the integration of organellar DNA fragments into nuclear genomes [84] and could resolve the conundrum of why group II introns did not persist in non-coding regions of eukaryotic genomes, where they are not subject to selective pressures caused by translational repression and NMD [13]. Given the inability of multiple group II introns that had inserted into protein-coding genes in an ancestral eukaryote to be cleanly excised simultaneously or to mutate readily into a form that could splice efficiently at low Mg^2+^ concentration, the evolutionary response was their degeneration into relatively unstructured spliceosomal introns that maintain conserved splice site and branch-point sequences. Reflecting their evolutionary origin, these conserved sequences are recognized by a common splicing apparatus consisting of snRNAs derived from group II intron domains that can now with the aid of proteins promote splicing in the low Mg^2+^ environment of the eukaryotic nucleus. More generally, our results suggest that differences in intracellular environment had a profound impact on the evolution of introns and gene expression mechanisms in bacteria and eukarya.

## Materials and Methods

### Mammalian cell lines and *E*. *coli* strains

Mammalian cells were grown in culture media supplemented with 10% fetal bovine serum (Gemini Biosystems), penicillin, and streptomycin at 37°C with 5% CO_2_ unless otherwise stated. HEK-293 (ATCC) and HEK-293 Flp-In cells (Invitrogen; Flp-In 293) were maintained in Dulbecco's Modified Eagle Medium (DMEM; Invitrogen) supplemented with glutaMAX (Invitrogen), and hygromycin B. HeLa cells were maintained in Eagle’s Minimum Essential Medium (EMEM; Invitrogen). COS-7 cells were maintained in DMEM. Antibiotics were added at the following concentrations: ampicillin (100 μg/ml), carbenicillin (150 μg/ml), hygromycin B (50–100 μg/ml), penicillin (1,000 U/ml), streptomycin (1,000 μg/ml), and tetracycline (15 μg/ml). Transfection reagents were: Fugene 6 (Roche), Lipofectamine 2000 (Life Technologies), Polyfect (Qiagen), and polyethylenimine (PEI; 40,000 linear molecular weight; Polysciences Inc).

*E*. *coli* HMS174(λDE3) (Novagen) was used for the selection of recipient plasmids after retrohoming of the Ll.LtrB intron into the plasmid target site in human cells. Electrocompetent HMS174(λDE3) were generated as described [10,85] and had a transformation efficiency of >2 x 10^10^ colony-forming units measured using pUC19 plasmid. *E*. *coli* strain DH5α was used for cloning.

### Recombinant plasmids

Plasmid phLtrA is a derivative of pAAV (Stratagene) that expresses a human codon-optimized LtrA ORF (hLtrA; see below) with a 3X myc tag and SV40-NLS fused to its C-terminus. The hLtrA ORF is cloned behind a CMV promoter and followed by a human growth hormone polyadenylation signal. Plasmid phLtrA1 is an earlier hLtrA expression plasmid in which the human codon-optimized LtrA ORF with an SV40-NLS fused to its C-terminus is cloned behind a CMV promoter in a pIRES vector (Clontech). The LtrA ORF contains a small artificial spliceosomal intron, subsequently found to be unnecessary for hLtrA expression, inserted after the start codon and is followed by an SV40 polyadenylation signal. pLtrA is the same except with the native non-codon optimized LtrA ORF.

Plasmid pLl.LtrB contains an Ll.LtrB-ΔORF intron RNA (Ll.LtrB-ΔD4(B1-B3) [86]) cloned downstream of a T7 promoter in a TOPO2.1 vector (Invitrogen). Variants of this plasmid include pLl.LtrB-GFP in which Ll.LtrB intron and flanking exons interrupts the GFP ORF at position 386; pGFP, which contains a T7-driven GFP ORF with the 35-nt ligated exon sequence that would result from Ll.LtrB intron splicing inserted at position 386; and pLl.LtrB-HPRT and pLl.LtrB-CCR5 in which the wild-type Ll.LtrB-ΔD4(B1-B3) intron is replaced by one that has been retargeted to insert in the mouse *hprt* gene (position 115; [45]) or human *CCR5* gene (position 332; [6]), respectively; pLl.LtrB-T7 is a derivative of Ll.LtrB-ΔD4(B1-B3) that contains a minimal T7 promoter in DIVb (positions 627–646); and pLl.LtrB-stuffer is a derivative that lacks the Ll.LtrB intron and was used for library construction.

Plasmid pCMV-BFP, pCMV-BFP-E1E2, and pCMV-BFP-Ll.LtrB contain the blue fluorescent protein (BFP) ORF without or with the *ltrB* exons 1 and 2 or the Ll.LtrB-ΔD4(B1-B3) intron flanked by *ltrB* exons 1 and 2 interrupting the ORF after the start codon cloned in pcDNA5FRT (Invitrogen).

Plasmid pT7-NLS contains the T7 RNA polymerase (T7 RNAP) ORF with an N-terminal SV40-NLS cloned behind a CMV promoter in pAAV vector (Agilent), and pT7 is the same plasmid containing the T7 RNAP ORF without a NLS.

Recipient plasmid pFRT contains a wild-type Ll.LtrB target site (positions -30 to + 15 from the intron-insertion site) inserted into the Flp-In recombinase site of pcDNA5/FRT (Life Technologies). The target site region is identical to that inserted into the HEK-293 Flp-In genome. Recipient plasmid pBRRQ is a derivative of pBRR-Tet [6] and contains a wild-type Ll.LtrB target site (positions -30 to +15 from the intron-insertion site) flanked by sequences with *T*_*m*_ values optimized for qPCR (S1 Table) cloned upstream of a promoter-less *tet* ^R^ gene. Recipient plasmid pBRR-CCR5 is identical to pBBRQ except for containing the CCR5 targetron insertion site (positions -30 to +15 from the intron insertion site). All recipient plasmids carry an *amp* ^R^ marker.

### Codon optimization of the LtrA ORF

The human codon optimized LtrA sequence was generated from overlapping oligonucledotides by assembly PCR [65]. Oligonucleotides containing hLtrA sequence were synthesized by HHMI/Keck Oligonucleotide Synthesis Facility (Yale) and PCR reactions were carried out by using Vent DNA polymerase (New England Biolabs), high annealing temperatures (58–60°C), and manual hot start–*i*.*e*., adding Vent DNA polymerase after sample temperature reached 94°C). PCR products were gel-purified and digested with EcoRI and XbaI or HindIII and XbaI, then cloned into pKSBluescript (Agilent) to form pKS-hLtrA and confirmed by sequencing. The assembled ORF was re-cloned into a pIRES vector (Clontech) to generate phLtrA1.

### Cytotoxicity analysis

HEK-293 cells were seeded at equal density into 96-well white plates (Corning), allowed to grow out, and transfected using Fugene 6 (Roche) according to manufacturer’s recommendations. After 48 h in culture, cytotoxicity analysis was carried out using the CellTiter-Glo direct lysis kit (Promega) according to manufacturer instructions. Luciferase activity was measured on a Mithras Multimode Platereader (Berthold). Trypan blue staining was performed by mixing 10 μl of cells with 10 μl of trypan blue solution (0.4%; Invitrogen) and then counting stained and unstained cells on a hemacytometer.

### Immunoblots and immunofluorescence

For immunoblotting, cells were collected and boiled in 1x Laemmli gel buffer for 5 min. After pelleting insoluble material by centrifugation in a microfuge for 2 min at top speed, the protein samples prepared from the same number of cells were run in 8% polyacrylamide/0.1% SDS gel, which was then blotted to a nitrocellulose membrane using a Hoefer SemiPhor blotter (Amersham). Anti-LtrA antibody [49] was used at 1:1,000 dilution, and goat anti-rabbit secondary antibody (Pierce) was used at 1:60,000 dilution, both at room temperature. After developing the immunoblot, the membrane was stained with AuroDye to confirm even loading.

For immunofluorescence, cells were washed twice with phosphate buffered saline (PBS) and then fixed in 2% paraformaldehyde for 30 min at room temperature. After three more washes with PBS, cells were permeabilized by incubating in 0.5% Triton X-100 in PBS for 15 min, followed by three washes with PBS containing 0.2% Tween 20 (PBST). Blocking was achieved by incubating the permeabilized cells with 10% normal goat serum and 1% BSA in PBST for 1 h. Primary antibody was pre-incubated with untransfected cell lysate (prepared by sonication) to deplete nonspecific antibodies and then incubated with cells at 1:5,000 dilution in blocking buffer for 1 h at 4°C. After four 5-min washes in PBST containing 0.1 M NaCl, cells were incubated with 1:100 dilution of goat anti-rabbit antibody conjugated with fluorescein in blocking buffer for 1 h, washed with PBST containing 0.1 M NaCl five times for 5 min each time, incubated with 2 μg/ml Hoechst dye for 10 min, and washed twice with PBS. Cells were mounted and observed under a fluorescence microscope (Olympus CKX41).

### Assay of reverse transcriptase activity of expressed LtrA protein in nuclear lysates

HEK-293 cells were grown to confluence, washed with PBS, blown off the dishes with ice-cold hypotonic buffer (10 mM HEPES, 10 mM KCl, 1 ml/100 mm dish), and incubated on ice for 15 min. Cells were broken by 15 strokes of a Dounce homogenizer. Nuclei were collected by centrifugation at 800 x g for 5 min at 4°C and then resuspended in the residual buffer in the same tube. After 3 cycles of freezing and thawing, chromosomal DNA was sheared by repeated pipetting, and 5 μl of the solution was used for each reaction. RT assays with Ll.LtrB/E2+10 substrate were carried out as described [47,49] in 10 μl of reaction medium containing 5 μl lysate, 40 nM Ll.LtrB template, 400 nM E2+10 primer, 450 mM NaCl, 5 mM MgCl_2_, 40 mM Tris-HCl, pH 7.5 plus 10 μCi [α-^32^P]dTTP (3,000 Ci/mmol; New England Nuclear) and 0.2 mM of each dNTP. The Ll.LtrB/E2+10 substrate consists of Ll.LtrB RNA (an *in vitro* transcript containing the Ll.LtrB-∆ORF intron and flanking exons) with a 20-mer DNA primer (E2+10) annealed to a position in the 3’ exon that corresponds to that of the cleaved bottom strand normally used as the primer for target DNA-primed reverse transcription of the intron RNA during retrohoming. Reactions were initiated by adding dNTPs and incubated at 30°C for 30 min. Incorporation of [α-^32^P]dTTP was measured by spotting onto DE81 paper (Whatman) and counting Cherenkov radiation in a scintillation counter (LS6500, Beckman).

### siRNA knockdown experiments

UPF1 and scramble siRNAs (Dharmacon) were transfected into ~60% confluent HeLa or HEK-293 cells 24 h prior to transfection of BFP- or GFP-containing plasmids. UPF1 levels were measured in equivalent amount of proteins from crude cell lysates via SDS-PAGE (4–12% polyacrylamide gradient gel) and immunoblotting using a Trans-Blot Turbo system (Bio-Rad) to blot the gel to a nitrocellulose membrane, which was then probed with an anti-UPF1 antibody (ab10510; Abcam). Plasmid and siRNA transfections were carried out using Dharmafect as described [87].

### RT-PCR, RT-qPCR, and Taqman qPCR

For analysis of transcript levels and splicing via RT-qPCR and RT-PCR, respectively, RNA was purified from transfected cells using the ZR RNA Miniprep Kit (Zymo). 1 μg of each RNA sample was treated with DNase I (Invitrogen) at 37°C for 1 h to remove DNA and then converted to cDNA with a SuperScript III reverse transcriptase kit (Invitrogen) according to manufacturer’s recommendations. RT-PCR was carried out with GC-rich Phusion polymerase mastermix (New England Biolabs) under standard conditions, unless otherwise indicated. RT-qPCR was carried out using Power SYBR Green Master Mix (ABI) on an Applied Biosystems Viia7 system in 96-well format under standard conditions. For the CMV-BFP cassettes, the primers were pAAV MCSfw 5’ TCTTATCTTCCTCCCACAGCTCCT and GFP-L qPCRrev 5’ TCGTCCTTGAAGAAGATGGTG, and for the T7-GFP cassette, the primers were pTOPOsplicinginfw 5’ TGTCTTCTTGACGAGCATTCC and pTOPOsplicinginrev 5’ TAGGTCAGGGTGGTCACGA.

Retrohoming of the Ll.LtrB intron in mammalian cells was assayed by Taqman qPCR using an Applied Biosystems Viia7 system in 384-well format using Taqman probes (Life Technologies). Reactions were performed in technical triplicate in 10-μl volumes for 35 (plasmid) or 40 (genomic) cycles using Taqman PCR universal mastermix (Applied Biosystems) under standard conditions. Standard curves for quantitation used four 10-fold dilutions of either pBRRQ or pFRT plasmid containing an integrated Ll.LtrB intron and had >90% efficiency across the range of concentrations used. Standard curve plasmids were quantified using a Qubit system (Life Technologies). Standard curve dilutions were buffered with 10 ng/μl phage lambda DNA carrier. The primer/probe sets are shown in S3 Table.

### Retrohoming of Ll.LtrB in HEK-293 Flp-In cells

HEK-293 Flp-In cells (Invitrogen) contain a FRT recombinase site in a decondensed region of the genome. A single copy of the wild-type Ll.LtrB insertion site (position -30 to +15 from the intron-insertion site) was recombined into the FRT site genomic locus according to manufacturer's recommendations. For retrohoming experiments, HEK-293 Flp-In cells containing the Ll.LtrB target site were seeded in multi-well culture plates (Corning) 24 h prior to transfection to reach a confluency of 60–80% on the day of transfection. Cells were dissociated using Stem Pro Accutase (Invitrogen), and cell counting was performed with a hemocytometer or using the Scepter system (Millipore).

For genomic targeting experiments, the Ll.LtrB intron expression plasmids, pLl.LtrB, pT7-NLS, and phLtrA were transfected at 276 ng each with 2.76 μg branched polyethyleneimine (PEI) (Polysciences, Inc) per well in a 12-well culture plate for 24 h. For plasmid targeting experiments, recipient plasmid pFRT or pBRRQ was included at 276 ng per well in addition to the above three plasmids. After 24 h, the media was removed and replaced with growth medium supplemented with MgCl_2_ or other Mg^2+^ salts for an additional 24 h unless otherwise specified. The next day, when the cells were typically 80–90% confluent, non-adherent cells were removed by vigorously rinsing with PBS three times, and adherent cells were collected into a 1.5-ml snap-tube unless otherwise specified. Total DNA was extracted from cell pellets with a Qiagen Blood and Tissue kit with an RNase step or the ZR-genomic miniprep kit (Zymo research) according to manufacturer's recommendations. In plasmid targeting experiments, plasmids were extracted from cells using alkaline lysis with the Wizard SV-miniprep system (Promega) or total DNA using the ZR-genomic miniprep kit (Zymo Research). Experiments typically used three wells that had been independently seeded and transfected in parallel for determination of SEMs. Biological replicates were performed on separate days and reported with SDs.

### Ll.LtrB mutant library generation

pLl.LtrB-T7 mutant libraries for each selection cycle were generated by PCR with Mutazyme II (Stratagene) according to the manufacturer’s recommendations for 3 mutations per kb. Approximately 200 ng Ll.LtrB DNA template was mutagenized in a 50-μl PCR with primers 309S 5’- CACATCCATAACGTGCGCC and 308A 5’- TAATTGCTAGCCGGCCGCATTAAAAATGATATG for 30 cycles, and then re-amplified to obtain a higher yield using Phusion polymerase (New England Biolabs). The PCR product was purified from an agarose gel stained with Sybr gold (Invitrogen) under blue-light illumination and then digested overnight with AatII and NheI-HF (New England Biolabs). After purification, 750 ng of the insert was ligated to 1 μg of linearized and dephosphorylated pLl.LtrB-stuffer for 2 h at room temperature in a volume of 400 μl using T4 DNA ligase (4,000 units; New England Biolabs). The ligation mix was purified and concentrated to a volume of 6 μl using a Zymo clean and concentrator column and then electroporated into 100 μl *E*. *coli* MegaXDH10B cells (Invitrogen) with total transformants typically reaching >2 x 10^8^. The resulting library was purified by using an Endotoxin-free MiniKit II (Omega Biosciences) and transfected into HEK-293 Flp-In cells for both targeting and selection experiments.

### *In vivo* selections for retrohoming of the Ll.LtrB intron in HEK-293 cells

*In vivo* selections in HEK-293 cells were done using a modification of a previously described *E*. *coli* plasmid-based retrohoming assay in which a group II intron with a phage T7 promoter inserted in DIVb integrates into a target site cloned in a recipient plasmid upstream of a promoterless *tet* ^R^ gene, thereby activating that gene [10,36]. HEK-293 cells were transfected with plasmids for the hybrid Pol II/T7 expression system (Fig 1), with pLl.LtrB replaced with pLl.LtrB-T7, which contains a minimal T7 promoter in DIVb, and pBRRQ, which contains an Ll.LtrB target site cloned upstream of a promoter-less *tet* ^*R*^ gene. After 24 h, plasmids were isolated from transfected cells by alkaline lysis using the Wizard SV plasmid miniprep kit (Promega). An aliquot was diluted and used for Taqman qPCR and the rest was concentrated to 6 μl using a Zymo clean and concentrator column. The concentrated plasmid was electroporated into 100 μl of electrocompetent *E*. *coli* HMS174(λDE3) cells, which were then plated onto LB-agar plates containing tetracycline (15 μg/ml) and grown for 2 days. The resulting colonies were pooled, and the Tet^R^ plasmids were isolated by alkaline lysis using a Wizard SV miniprep kit (Promega). Ll.LtrB introns that had successfully retrohomed into the Tet^R^-recipient plasmids were PCR amplified by 21 cycles of PCR with or without mutagenesis as described above using primers that flank the integration site (primers 200S and 269A; S3 Table), and the PCR product was isolated from an agarose gel and used to generate a library for the next round of selection.

### Construction of a synthetic shuffling library

Assembly PCR was used to generate the synthetically shuffled library [65]. Briefly, multiple 80-120-mer oligonucleotides spanning the length of the intron and containing the randomized or doped positions of interest and complementary overlaps with a *T*_*m*_ of ~55°C were synthesized at the Center for Systems and Synthetic Biology at UT-Austin. For each intron library, the assembly PCR was done with a 500-ng equimolar mix of oligonucleotides for 25 cycles under standard conditions in 50 μl of Phusion PCR mastermix. A 5-μl aliquot was placed in 300 μl of Phusion PCR mix with forward and reverse primers that synthesize the full-length intron and run for an additional 25 cycles. The full-length product was purified by electrophoresis in an agarose gel and used to construct libraries in pLl.LtrB, as described above.

### High-throughput sequencing and computational analysis

Libraries for Pacific Biosciences RS circular consensus sequencing (CCS) were generated according to manufacture's recommendations for A-tailed inserts, and sequencing was performed at the Johns Hopkins University Medical School deep sequencing and microarray core facility. Inserts for PacBio sequencing were generated directly from pooled Tet^R^-positive plasmids isolated after directed evolution cycles by digesting >50 μg of plasmid DNA with AatII and EcoRI-HF (New England Biolabs) at sites 37-nt upstream and 16-nt downstream of the Ll.LtrB-integration site, respectively, and then purifying the resulting restriction fragment in a 1% agarose gel under blue light using Sybr Gold staining. To assess the sequencing error-rate for the PacBio CCS, we sequenced the wild-type intron and determined the number of substitutions, insertion, and deletion errors. With three rolling-circle sequencing passes of the intron, the substitution error rate was <0.01%. The insertion and deletion (indel) rates were 0.21 and 0.07% respectively, and these occurred predominantly at homopolymeric regions.

Sequence reads were filtered to remove reads that did not reach at least three circular passes. Raw sequence reads in the FastQ file format were aligned to the wild-type Ll.LtrB reference sequence using Mosaik Aligner 1.0 (https://code.google.com/p/mosaik-aligner/) and text files were extracted using the Tablet browser [88]. Insertion gaps were removed using a Perl script, Gapstreeze, available online at (http://www.hiv.lanl.gov/content/sequence/GAPSTREEZE/gap.html), and reads containing deletion-errors were removed. Aligned sequences were then analyzed for nucleotide variation using a Perl script courtesy of Dr. Scott Hunicke-Smith (UT-Austin). All other data analysis, including calculation of nucleotide frequencies and analysis of co-variations was performed using Unix shell scripts, including grep, cut, uniq, sort, and awk.

Standard linkage disequilibrium was calculated as *D* = (*P*_*AB*_ x *P*_*ab*_)-(*P*_*Ab*_ x *P*_*aB*_), where *P*_*AB*_ is the frequency at which the mutations occur together, *P*_*Ab*_ and *P*_*aB*_ are the mutations occurring independently, and *P*_*ab*_ the frequency at which neither occurred. The normalized linkage disequilibrium (*D'*) was calculated by dividing positive *D* values by the theoretical maximum co-occurrence and negative *D* values by a theoretical minimum co-occurrence based on the observed individual frequencies in the population. The significance of these values was measured with the *r* ^*2*^ value (the square of the correlation coefficient) calculated as *r* ^*2*^ = *D* ^*2*^ */P*_*a*_ *P*_*b*_ *P*_*A*_ *P*_*B*_, and *χ* ^*2*^ which is *r* ^*2*^ multiplied by the number of sequences analyzed [77].

### Data availability

The Pacific Biosciences sequencing data are available at the NCBI SRA database (Biosample accession numbers: SAMN03342363, SAMN03342364, SAMN03342365 and SAMN03342366). The hLtrA sequence is available from NCBI Genbank (accession number KP851976). The primary data underlying the Figures are available in S1 Data.

## Supporting Information

S1 Fig Predominantly nuclear expression of the group II intron RNA is not required for retrohoming into genomic or plasmid target sites. (A) Genomic and (B) plasmid retrohoming assays were done in HEK-293 in culture medium supplemented with 80 mM MgCl_2_ using the three-plasmid Ll.LtrB group II intron expression system with T7 RNAP expressed with or without an appended SV40 NLS. Negative controls were assays in which magnesium was not added to the cell culture medium. Retrohoming efficiencies were measured in adherent cells by the Taqman qPCR assays diagrammed in Fig 5. Blue and red bars show frequencies of 5’- and 3’-integration junctions, respectively, relative to copies of a sequence within the hygromycin-resistance gene (*hyg* ^R^) adjacent to the retrohoming site. The bar graphs show the average for three separate transfections with the error bars indicating the SEM.
(PDF)

S2 Fig PCR and sequencing of full-length group II intron integrations into a plasmid target site in human cells. (A) PCR analysis. Retrohoming assays were done in HEK-293 Flp-In cells transfected with Ll.LtrB expression plasmids plus recipient plasmid pFRT with or without 80 mM Mg^2+^ added to the culture medium, as described in Fig 5. PCR was done on total DNA extracted from the cells using primers 176S 5’- CATCCATAACGTGCGCC and 269A; S3 Table). The upstream primer anneals to the 5’-integration junction (positions -10 to +7), and the downstream primer anneals to a sequence 28-nt downstream of the 3’ integration junction. Each of the samples shown is from a separate transfection. (B) Sanger sequencing of a PCR product from (A), confirming the correct 3’- integration junction.
(PDF)

S3 Fig Effects of modified Mg^2+^ treatments on retrohoming and cell viability. (A) Retrohoming into the genomic target site and cell viability in HEK-293 cells in culture medium supplemented with different concentrations of MgCl_2_. The bar graph on the left shows the retrohoming frequency assayed by Taqman qPCR of 3’-integration junctions in DNA extracted from HEK-293 cells (adherent and non-adherent) transfected with the Ll.LtrB expression plasmids after incubation in medium containing the indicated Mg^2+^ concentration for 24 h. The (-) control indicates absence of both phLtrA protein and MgCl_2_. Values are the mean for three separate transfections on the same day, with the error bar indicating the SEM. The right shows a plot of cells viability prior to DNA extraction as determined by trypan blue staining. Reattachment refers to the percentage of cells that re-adhered in 24 h after trypsin treatment and subsequent re-plating. (B) Time course for retrohoming into the genomic site in HEK-293 cells in the presence of 80 mM MgCl_2_. The bar graphs show retrohoming frequencies assayed by Taqman qPCR of 3’-integration junctions in DNA extracted from HEK-293 cells (adherent and non-adherent) transfected with the Ll.LtrB expression plasmids after incubation in culture medium supplemented with 80 mM Mg^2+^ for different times. Values are the mean for three separate transfections on the same day, with the error bars indicating the SEM. (C) Retrohoming into the genomic site in HEK-293 cells in culture medium supplemented with different Mg^2+^ salts. The top and bottom bar graphs compare retrohoming frequencies assayed by Taqman qPCR of 3’-integration junctions in adherent versus non-adherent HEK-293 cells after 24 h in culture medium supplemented with 40 or 80 mM of different Mg^2+^ salts: Cl, MgCl_2_; Su, MgSO_4_; Ac, MgOAc; Gl, Mg-glutamate; As, Mg-aspartate; and m-80, equimolar MgCl_2_, MgSO_4_, Mg-glutamate, and Mg-aspartate. The concentration used is indicated next to the counter ion abbreviation.
(PDF)

S4 Fig DV mutants selected for enhanced retrohoming in *E*. *coli* did not show increased retrohoming frequencies for genomic or plasmid target sites in HEK-293 cells. Ll.LtrB variants DV14 and DV20 with mutations in the distal stem of DV selected for enhanced retrohoming in Mg^2+^-deficient *E*. *coli* [36] were tested in parallel to the wild-type intron for retrohoming into (A) genomic or (B) plasmid target sites in HEK-293 cells with or without 80 mM MgCl_2_ added to the culture medium. Cells were transfected with phLtrA, pLl.LtrB, and pT7-NLS, and retrohoming was assayed by qPCR at 24 h after transfection. The assays done without extra Mg^2+^ added to the culture medium are denoted 0 mM MgCl_2_, and hLtrA(-) indicates a control done without transfection of phLtrA. The bar graphs show retrohoming frequencies assayed by Taqman qPCR of 5’- or 3’-integration junctions (blue and red, respectively) in adherent HEK-293 cells. Values are the mean for two or three separate transfections on the same day, with the error bars indicating the SEM.
(PDF)

S5 Fig A DV variant selected for enhanced retrohoming in *Xenopus laevis* oocyte nuclei did not show increased retrohoming frequencies into a genomic target site in HEK-293 cells. An Ll.LtrB variant (DV-XL7) with mutations in the distal stem of DV that result in four-fold increased retrohoming efficiency in *Xenopus laevis* oocytes [54] was tested in parallel with the wild-type intron and did not shown increased retrohoming frequencies into a genomic target site in HEK-293 cells with 80 mM MgCl_2_ added to the culture medium. The WT intron was tested without extra MgCl_2_ (No Mg^2+^) as a control. The bar graphs show retrohoming frequencies assayed by Taqman qPCR of 3’-integration junctions in DNA extracted from adherent HEK-293 cells transfected with the Ll.LtrB expression plasmids after incubation in medium containing the indicated Mg^2+^ concentration for 24 h. Values are the mean for two separate transfections on the same day, with the error bars indicating the SD.
(PDF)

S6 Fig Tet^R^ plasmids recovered after retrohoming of the Ll.LtrB introns in HEK-293 cells contain full-length integrated intron with the expected 5’- and 3’-integration junctions. (A) PCR amplification of full-length Ll.LtrB insertions from Tet^R^ recipient plasmids recovered by selection in *E*. *coli* from HEK-293 cells after retrohoming in the presence of 80 mM MgCl_2_ was done using primers 200S and 269A; S3 Table). The upstream primer anneals 32-nt upstream of the integration site, and the downstream primer anneals 28-nt downstream of the integration site. Approximately 50% of recovered plasmids contain the full-length intron integrations. The remainders are false positives. (B) Sanger sequencing of full-length intron integrations from a Tet^R^ plasmid recovered by selection in *E*. *coli*, confirming the expected 5-’ and 3’- integration junctions in the same PCR product.
(PDF)

S7 Fig Synthetic shuffling of prevalent positively selected Ll.LtrB mutations in HEK-293 cells over four selection cycles. Optimal combinations of mutations were identified by synthetic shuffling and selection in HEK-293 cells for four cycles with (A) 80 mM MgCl_2_ or (B) 40 mM MgCl_2_ added to the culture medium. The synthetic shuffling library was generated as described in Fig 9. The synthetically shuffled library was tested at the indicated MgCl_2_ concentration, and the wild-type intron was tested in parallel. 5'- and 3'-integration junctions were quantified by Taqman qPCR relative to *tet* ^*R*^ copies during the selection cycles and expressed relative to the retrohoming frequency of the wild-type intron assayed in parallel. Values are the mean for three separate transfections on the same day, with the error bars indicating the SEM.
(PDF)

S1 Table Top mutation combinations identified in the HEK-293 selections. The frequency refers to the percentage of reads with the indicated mutations and all other positions remaining wild type after selection rounds 8 and 12. By comparison, the average frequency of variants occurring only once was ~0.03–0.07% of the total sequencing reads for each library.
(DOCX)

S2 Table Standard linkage disequilibrium of mutations found in HEK-293 directed evolution round 8. The Table shows calculated values for standard linkage disequilibrium (*D*) and the normalized linkage disequilibrium (*D'*) between the highest frequency mutations in the HEK-293 cell selection at round 8 (see Materials and Methods). The value for *D* and *D'* can be positive or negative, indicating whether the combinations of mutations occur more or less frequently, respectively, than expected from the frequency of each mutation by itself. Values close to zero indicate linkage equilibrium between the two mutations. The *r* ^*2*^ and *Χ* ^*2*^ values indicate the significance of the disequilibrium, with higher numbers indicating greater significance.
(DOCX)

S3 Table Primers used for Taqman qPCR assays of Ll.LtrB retrohoming in human cells. Taqman probes and primers used for detecting retrohoming of the Ll.LtrB intron in HEK-293 cells. The *hyg* ^*R*^ target refers to the gene encoding hygromycin phosphotransferase, which confers hygromycin B resistance in the HEK-293 Flp-In cells. It is located upstream of the wild-type Ll.LtrB target site in the genomic FRT recombinase site. Taqman probes with 5'-FAM (6-carboxyfluorescien) and 3'-MGB (dihydrocyclopyrroloindole tripeptide major groove binder) were obtained from Applied Biosystems and those with 5'-FAM and 3'-BkFQ (Iowa Black FQ) from Integrated DNA Technologies.
(DOCX)

S1 Data Excel spreadsheet of primary data for Figs 1, 3–9, S1, S3-S5, and S7. (XLSX)

## References

[1] Lambowitz AM, Zimmerly S. Group II introns: mobile ribozymes that invade DNA. Cold Spring Harb Perspect Biol. 2011;3: a003616 10.1101/cshperspect.a003616 20463000 PMC3140690

[2] Zimmerly S, Hausner G, Wu X. Phylogenetic relationships among group II intron ORFs. Nucleic Acids Res. 2001;29: 1238–1250. 11222775 10.1093/nar/29.5.1238 PMC29734

[3] Martin W, Koonin EV. Introns and the origin of nucleus-cytosol compartmentalization. Nature. 2006;440: 41–45. 16511485 10.1038/nature04531

[4] Lambowitz AM, Belfort M (2015) Mobile bacterial group II introns at the crux of eukaryotic evolution. Microbiol. Spectrum, 3(1) MDNA3-0050-2014; and In: Craig NL, Gellert M, Lambowitz AM, Chandler M, Rice P, Sandmeyer S, editors (2015). Mobile DNA III. Washington DC: ASM Press. 10.1128/microbiolspec.MDNA3-0050-2014 PMC4394904 26104554

[5] Yang J, Zimmerly S, Perlman PS, Lambowitz AM. Efficient integration of an intron RNA into double-stranded DNA by reverse splicing. Nature. 1996;381: 332–335. 8692273 10.1038/381332a0

[6] Guo H, Karberg M, Long M, Jones JP, Sullenger B, Lambowitz AM. Group II introns designed to insert into therapeutically relevant DNA target sites in human cells. Science. 2000;289: 452–457. 10903206 10.1126/science.289.5478.452

[7] Karberg M, Guo H, Zhong J, Coon R, Perutka J, Lambowitz AM. Group II introns as controllable gene targeting vectors for genetic manipulation of bacteria. Nat Biotechnol. 2001;19: 1162–1167. 11731786 10.1038/nbt1201-1162

[8] Perutka J, Wang W, Goerlitz D, Lambowitz AM. Use of computer-designed group II introns to disrupt *Escherichia coli* DExH/D-box protein and DNA helicase genes. J Mol Biol. 2004;336: 421–439. 14757055 10.1016/j.jmb.2003.12.009

[9] Enyeart PJ, Mohr G, Ellington AD, Lambowitz AM. Biotechnological applications of mobile group II introns and their reverse transcriptases: gene targeting, RNA-seq, and non-coding RNA analysis. Mob DNA. 2014;5: 2 10.1186/1759-8753-5-2 24410776 PMC3898094

[10] Mastroianni M, Watanabe K, White TB, Zhuang F, Vernon J, Matsuura M et al Group II intron-based gene targeting reactions in eukaryotes. PLOS One. 2008;3: e3121 10.1371/journal.pone.0003121 18769669 PMC2518211

[11] Chalamcharla VR, Curcio MJ, Belfort M. Nuclear expression of a group II intron is consistent with spliceosomal intron ancestry. Genes Dev. 2010;24: 827–836. 10.1101/gad.1905010 20351053 PMC2854396

[12] Zerbato M, Holic N, Moniot-Frin S, Ingrao D, Galy A, Perea J. The brown algae Pl.LSU/2 group II intron-encoded protein has functional reverse transcriptase and maturase activities. PLOS One. 2013;8: e58263 10.1371/journal.pone.0058263 23505475 PMC3594303

[13] Doolittle WF. The trouble with (group II) introns. Proc Natl Acad Sci U S A. 2014;111: 6536–6537. 10.1073/pnas.1405174111 24757059 PMC4020116

[14] Peebles CL, Perlman PS, Mecklenburg KL, Petrillo ML, Tabor JH, Jarrell KA et al A self-splicing RNA excises an intron lariat. Cell. 1986;44: 213–223. 3510741 10.1016/0092-8674(86)90755-5

[15] Toor N, Keating KS, Taylor SD, Pyle AM. Crystal structure of a self-spliced group II intron. Science. 2008;320: 77–82. 10.1126/science.1153803 18388288 PMC4406475

[16] Marcia M, Pyle AM. Visualizing group II intron catalysis through the stages of splicing. Cell. 2012;151: 497–507. 10.1016/j.cell.2012.09.033 23101623 PMC3628766

[17] Robart AR, Chan RT, Peters JK, Rajashankar KR, Toor N. Crystal structure of a eukaryotic group II intron lariat. Nature. 2014;514: 193–197. 10.1038/nature13790 25252982 PMC4197185

[18] Carignani G, Groudinsky O, Frezza D, Schiavon E, Bergantino E, Slonimski PP. An mRNA maturase is encoded by the first intron of the mitochondrial gene for the subunit I of cytochrome oxidase in *S*. *cerevisiae* . Cell. 1983;35: 733–742. 6317200 10.1016/0092-8674(83)90106-x

[19] Matsuura M, Saldanha R, Ma H, Wank H, Yang J, Mohr G et al A bacterial group II intron encoding reverse transcriptase, maturase, and DNA endonuclease activities: biochemical demonstration of maturase activity and insertion of new genetic information within the intron. Genes Dev. 1997;11: 2910–2924. 9353259 10.1101/gad.11.21.2910 PMC316661

[20] Matsuura M, Noah JW, Lambowitz AM. Mechanism of maturase-promoted group II intron splicing. EMBO J. 2001;20: 7259–7270. 11743002 10.1093/emboj/20.24.7259 PMC125332

[21] Saldanha R, Chen B, Wank H, Matsuura M, Edwards J, Lambowitz AM. RNA and protein catalysis in group II intron splicing and mobility reactions using purified components. Biochemistry. 1999;38: 9069–9083. 10413481 10.1021/bi982799l

[22] Singh NN, Lambowitz AM. Interaction of a group II intron ribonucleoprotein endonuclease with its DNA target site investigated by DNA footprinting and modification interference. J Mol Biol. 2001;309: 361–386. 11371159 10.1006/jmbi.2001.4658

[23] Zimmerly S, Guo H, Perlman PS, Lambowitz AM. Group II intron mobility occurs by target DNA-primed reverse transcription. Cell. 1995;82: 545–554. 7664334 10.1016/0092-8674(95)90027-6

[24] Zimmerly S, Guo H, Eskes R, Yang J, Perlman PS, Lambowitz AM. A group II intron RNA is a catalytic component of a DNA endonuclease involved in intron mobility. Cell. 1995;83: 529–538. 7585955 10.1016/0092-8674(95)90092-6

[25] Smith D, Zhong J, Matsuura M, Lambowitz AM, Belfort M. Recruitment of host functions suggests a repair pathway for late steps in group II intron retrohoming. Genes Dev. 2005;19: 2477–2487. 16230535 10.1101/gad.1345105 PMC1257402

[26] Yao J, Truong DM, Lambowitz AM. Genetic and biochemical assays reveal a key role for replication restart proteins in group II intron retrohoming. PLOS Genet. 2013;9: e1003469 10.1371/journal.pgen.1003469 23637634 PMC3636086

[27] Sharp PA. “Five easy pieces”. Science. 1991;254: 663 1948046 10.1126/science.1948046

[28] Keating KS, Toor N, Perlman PS, Pyle AM. A structural analysis of the group II intron active site and implications for the spliceosome. RNA. 2010;16: 1–9. 10.1261/rna.1791310 19948765 PMC2802019

[29] Gordon PM, Sontheimer EJ, Piccirilli JA. Metal ion catalysis during the exon-ligation step of nuclear pre-mRNA splicing: extending the parallels between the spliceosome and group II introns. RNA. 2000;6: 199–205. 10688359 10.1017/s1355838200992069 PMC1369906

[30] Fica SM, Tuttle N, Novak T, Li NS, Lu J, Koodathingal P et al RNA catalyses nuclear pre-mRNA splicing. Nature. 2013;503: 229–234. 10.1038/nature12734 24196718 PMC4666680

[31] Fica SM, Mefford MA, Piccirilli JA, Staley JP. Evidence for a group II intron-like catalytic triplex in the spliceosome. Nat Struct Mol Biol. 2014;21: 464–471. 10.1038/nsmb.2815 24747940 PMC4257784

[32] Galej WP, Oubridge C, Newman AJ, Nagai K. Crystal structure of Prp8 reveals active site cavity of the spliceosome. Nature. 2013;493: 638–643. 10.1038/nature11843 23354046 PMC3672837

[33] Dlakic M, Mushegian A. Prp8, the pivotal protein of the spliceosomal catalytic center, evolved from a retroelement-encoded reverse transcriptase. RNA. 2011;17: 799–808. 10.1261/rna.2396011 21441348 PMC3078730

[34] Cavalier-Smith T. Intron phylogeny: a new hypothesis. Trends Genet. 1991;7: 145–148. 2068786

[35] Qu G, Dong X, Piazza CL, Chalamcharla VR, Lutz S, Curcio MJ et al RNA-RNA interactions and pre-mRNA mislocalization as drivers of group II intron loss from nuclear genomes. Proc Natl Acad Sci U S A. 2014;111: 6612–6617. 10.1073/pnas.1404276111 24722636 PMC4020058

[36] Truong DM, Sidote DJ, Russell R, Lambowitz AM. Enhanced group II intron retrohoming in magnesium-deficient *Escherichia coli* via selection of mutations in the ribozyme core. Proc Natl Acad Sci U S A. 2013;110: E3800–E3809. 10.1073/pnas.1315742110 24043808 PMC3791771

[37] Gregan J, Kolisek M, Schweyen RJ. Mitochondrial Mg^2+^ homeostasis is critical for group II intron splicing *in vivo* . Genes Dev. 2001;15: 2229–2237. 11544180 10.1101/gad.201301 PMC312778

[38] Gunther T. Concentration, compartmentation and metabolic function of intracellular free Mg^2+^ . Magnes Res. 2006;19: 225–236. 17402290

[39] Costa M, Fontaine JM, Loiseaux-de Goer S, Michel F. A group II self-splicing intron from the brown alga *Pylaiella littoralis* is active at unusually low magnesium concentrations and forms populations of molecules with a uniform conformation. J Mol Biol. 1997;274: 353–364. 9405145 10.1006/jmbi.1997.1416

[40] Haas J, Park EC, Seed B. Codon usage limitation in the expression of HIV-1 envelope glycoprotein. Curr Biol. 1996;6: 315–324. 8805248 10.1016/s0960-9822(02)00482-7

[41] Boshart M, Weber F, Jahn G, Dorsch-Hasler K, Fleckenstein B, Schaffner W. A very strong enhancer is located upstream of an immediate early gene of human cytomegalovirus. Cell. 1985;41: 521–530. 2985280 10.1016/s0092-8674(85)80025-8

[42] Cui X. RNA/protein interactions during group II intron splicing and toward group II intron targeting in mammalian cells. Ph.D. Dissertation. The University of Texas at Austin. 2006. Available: http://repositories.lib.utexas.edu

[43] Brisson M, He Y, Li S, Yang JP, Huang L. A novel T7 RNA polymerase autogene for efficient cytoplasmic expression of target genes. Gene Ther. 1999;6: 263–270. 10435111 10.1038/sj.gt.3300827

[44] Yarovoi SV, Pederson T. Human cell lines expressing hormone regulated T7 RNA polymerase localized at distinct intranuclear sites. Gene. 2001;275: 73–81. 11574154 10.1016/s0378-1119(01)00652-7

[45] Hanson JH. DNA target site recognition and toward gene targeting in mammalian cells by the Ll.LtrB group II intron RNP. Ph.D. Dissertation. The University of Texas at Austin. 2013. Available: http://repositories.lib.utexas.edu

[46] Nisa-Martinez R, Laporte P, Jimenez-Zurdo JI, Frugier F, Crespi M, Toro N. Localization of a bacterial group II intron-encoded protein in eukaryotic nuclear splicing-related cell compartments. PLOS One. 2013;8: e84056 10.1371/journal.pone.0084056 24391881 PMC3877140

[47] San Filippo J, Lambowitz AM. Characterization of the C-terminal DNA-binding/DNA endonuclease region of a group II intron-encoded protein. J Mol Biol. 2002;324: 933–951. 12470950 10.1016/s0022-2836(02)01147-6

[48] Nicholson P, Yepiskoposyan H, Metze S, Zamudio Orozco R, Kleinschmidt N, Muhlemann O. Nonsense-mediated mRNA decay in human cells: mechanistic insights, functions beyond quality control and the double-life of NMD factors. Cell Mol Life Sci. 2010;67: 677–700. 10.1007/s00018-009-0177-1 19859661 PMC11115722

[49] Cui X, Matsuura M, Wang Q, Ma H, Lambowitz AM. A group II intron-encoded maturase functions preferentially in cis and requires both the reverse transcriptase and X domains to promote RNA splicing. J Mol Biol. 2004;340: 211–231. 15201048 10.1016/j.jmb.2004.05.004

[50] Carapuca E, Azzoni AR, Prazeres DM, Monteiro GA, Mergulhao FJ. Time-course determination of plasmid content in eukaryotic and prokaryotic cells using real-time PCR. Mol Biotechnol. 2007;37: 120–126. 17914172 10.1007/s12033-007-0007-3

[51] Lam AP, Dean DA. Progress and prospects: nuclear import of nonviral vectors. Gene Ther. 2010;17: 439–447. 10.1038/gt.2010.31 20200566 PMC4084667

[52] Gilbert N, Lutz S, Morrish TA, Moran JV. Multiple fates of L1 retrotransposition intermediates in cultured human cells. Mol Cell Biol. 2005;25: 7780–7795. 16107723 10.1128/MCB.25.17.7780-7795.2005 PMC1190285

[53] Charras GT. A short history of blebbing. J Microsc. 2008;231: 466–478. 10.1111/j.1365-2818.2008.02059.x 18755002

[54] Truong DM. Mobile group II intron: host factors, directed evolution, and gene targeting in human cells. Ph.D. Dissertation. The University of Texas at Austin. 2014. Available: http://repositories.lib.utexas.edu

[55] Travers KJ, Chin CS, Rank DR, Eid JS, Turner SW. A flexible and efficient template format for circular consensus sequencing and SNP detection. Nucleic Acids Res. 2010;38: e159 10.1093/nar/gkq543 20571086 PMC2926623

[56] Lahr DJ, Katz LA. Reducing the impact of PCR-mediated recombination in molecular evolution and environmental studies using a new-generation high-fidelity DNA polymerase. Biotechniques. 2009;47: 857–866. 10.2144/000113219 19852769

[57] Shao W, Boltz VF, Spindler JE, Kearney MF, Maldarelli F, Mellors JW et al Analysis of 454 sequencing error rate, error sources, and artifact recombination for detection of low-frequency drug resistance mutations in HIV-1 DNA. Retrovirology. 2013;10: 18 10.1186/1742-4690-10-18 23402264 PMC3599717

[58] Singh RN, Saldanha RJ, D’Souza LM, Lambowitz AM. Binding of a group II intron-encoded reverse transcriptase/maturase to its high affinity intron RNA binding site involves sequence-specific recognition and autoregulates translation. J Mol Biol. 2002;318: 287–303. 12051838 10.1016/S0022-2836(02)00054-2

[59] Watanabe K, Lambowitz AM. High-affinity binding site for a group II intron-encoded reverse transcriptase/maturase within a stem-loop structure in the intron RNA. RNA. 2004;10: 1433–1443. 15273321 10.1261/rna.7730104 PMC1370629

[60] Mohr G, Smith D, Belfort M, Lambowitz AM. Rules for DNA target-site recognition by a lactococcal group II intron enable retargeting of the intron to specific DNA sequences. Genes Dev. 2000;14: 559–573. 10716944 PMC316424

[61] Chapman KA, Burgess RR. Construction of bacteriophage T7 late promoters with point mutations and characterization by *in vitro* transcription properties. Nucleic Acids Res. 1987;15: 5413–5432. 3299271 10.1093/nar/15.13.5413 PMC305969

[62] Sandig V, Lieber A, Bahring S, Strauss M. A phage T7 class-III promoter functions as a polymerase II promoter in mammalian cells. Gene. 1993;131: 255–259. 8406019 10.1016/0378-1119(93)90302-j

[63] Lieber A, Sandig V, Strauss M. A mutant T7 phage promoter is specifically transcribed by T7-RNA polymerase in mammalian cells. Eur J Biochem. 1993;217: 387–394. 8223577 10.1111/j.1432-1033.1993.tb18257.x

[64] Ness JE, Kim S, Gottman A, Pak R, Krebber A, Borchert TV et al Synthetic shuffling expands functional protein diversity by allowing amino acids to recombine independently. Nat Biotechnol. 2002;20: 1251–1255. 12426575 10.1038/nbt754

[65] Stemmer WP, Crameri A, Ha KD, Brennan TM, Heyneker HL. Single-step assembly of a gene and entire plasmid from large numbers of oligodeoxyribonucleotides. Gene. 1995;164: 49–53. 7590320 10.1016/0378-1119(95)00511-4

[66] Popp MW, Maquat LE. Organizing principles of mammalian nonsense-mediated mRNA decay. Annu Rev Genet. 2013;47: 139–165. 10.1146/annurev-genet-111212-133424 24274751 PMC4148824

[67] Rubin H. The logic of the Membrane, Magnesium, Mitosis (MMM) model for the regulation of animal cell proliferation. Arch Biochem Biophys. 2007;458: 16–23. 16750508 10.1016/j.abb.2006.03.026

[68] Cousineau B, Smith D, Lawrence-Cavanagh S, Mueller JE, Yang J, Mills D et al Retrohoming of a bacterial group II intron: mobility via complete reverse splicing, independent of homologous DNA recombination. Cell. 1998;94: 451–462. 9727488 10.1016/s0092-8674(00)81586-x

[69] Strick R, Strissel PL, Gavrilov K, Levi-Setti R. Cation-chromatin binding as shown by ion microscopy is essential for the structural integrity of chromosomes. J Cell Biol. 2001;155: 899–910. 11739403 10.1083/jcb.200105026 PMC2150894

[70] Kubo S, Seleme MC, Soifer HS, Perez JL, Moran JV, Kazazian HHJ et al L1 retrotransposition in nondividing and primary human somatic cells. Proc Natl Acad Sci U S A. 2006;103: 8036–8041. 16698926 10.1073/pnas.0601954103 PMC1472425

[71] Kinsey JA. Transnuclear retrotransposition of the Tad element of *Neurospora* . Proc Natl Acad Sci U S A. 1993;90: 9384–9387. 8415711 10.1073/pnas.90.20.9384 PMC47572

[72] Goodwin TJ, Ormandy JE, Poulter RT. L1-like non-LTR retrotransposons in the yeast *Candida albicans* . Curr Genet. 2001;39: 83–91. 11405100 10.1007/s002940000181

[73] Zhuang F, Mastroianni M, White TB, Lambowitz AM. Linear group II intron RNAs can retrohome in eukaryotes and may use nonhomologous end-joining for cDNA ligation. Proc Natl Acad Sci U S A. 2009;106: 18189–18194. 10.1073/pnas.0910277106 19833873 PMC2775298

[74] White TB, Lambowitz AM. The retrohoming of linear group II intron RNAs in *Drosophila melanogaster* occurs by both DNA ligase 4-dependent and-independent mechanisms. PLOS Genet. 2012;8: e1002534 10.1371/journal.pgen.1002534 22359518 PMC3280974

[75] Suzuki J, Yamaguchi K, Kajikawa M, Ichiyanagi K, Adachi N, Koyama H et al Genetic evidence that the non-homologous end-joining repair pathway is involved in LINE retrotransposition. PLOS Genet. 2009;5: e1000461 10.1371/journal.pgen.1000461 19390601 PMC2666801

[76] Chapman KB, Boeke JD. Isolation and characterization of the gene encoding yeast debranching enzyme. Cell. 1991;65: 483–492. 1850323 10.1016/0092-8674(91)90466-c

[77] Hayden EJ, Ferrada E, Wagner A. Cryptic genetic variation promotes rapid evolutionary adaptation in an RNA enzyme. Nature. 2011;474: 92–95. 10.1038/nature10083 21637259

[78] Hayden EJ, Wagner A. Environmental change exposes beneficial epistatic interactions in a catalytic RNA. Proc Biol Sci. 2012;279: 3418–3425. 10.1098/rspb.2012.0956 22719036 PMC3396916

[79] Gaj T, Gersbach CA, Barbas CF. ZFN, TALEN, and CRISPR/Cas-based methods for genome engineering. Trends Biotechnol. 2013;31: 397–405. 10.1016/j.tibtech.2013.04.004 23664777 PMC3694601

[80] Mali P, Esvelt KM, Church GM. Cas9 as a versatile tool for engineering biology. Nat Methods. 2013;10: 957–963. 10.1038/nmeth.2649 24076990 PMC4051438

[81] Lusk JE, Williams RJ, Kennedy EP. Magnesium and the growth of *Escherichia coli* . J Biol Chem. 1968;243: 2618–2624. 4968384

[82] Romani AM. Magnesium homeostasis in mammalian cells. Front Biosci. 2007;12: 308–331. 17127301 10.2741/2066

[83] Johnson EE, Wessling-Resnick M. Iron metabolism and the innate immune response to infection. Microbes Infect. 2012;14: 207–216. 10.1016/j.micinf.2011.10.001 22033148 PMC3270215

[84] Mishmar D, Ruiz-Pesini E, Brandon M, Wallace DC. Mitochondrial DNA-like sequences in the nucleus (NUMTs): insights into our African origins and the mechanism of foreign DNA integration. Hum Mutat. 2004;23: 125–133. 14722916 10.1002/humu.10304

[85] Sambrook J, Russell DW. Transformation of *E*. *coli* by electroporation. CSH Protoc. 2006;2006:10.1101/pdb.prot3933 22485378

[86] Wank H, SanFilippo J, Singh RN, Matsuura M, Lambowitz AM. A reverse transcriptase/maturase promotes splicing by binding at its own coding segment in a group II intron RNA. Mol Cell. 1999;4: 239–250. 10488339 10.1016/s1097-2765(00)80371-8

[87] Choi VW, Asokan A, Haberman RA, Samulski RJ. Production of recombinant adeno-associated viral vectors for *in vitro* and *in vivo* use. Curr Protoc Mol Biol. 2007;Chapter 16: Unit 16.25. 10.1002/0471142727.mb1625s78 18265393

[88] Milne I, Bayer M, Cardle L, Shaw P, Stephen G, Wright F et al Tablet—next generation sequence assembly visualization. Bioinformatics. 2010;26: 401–402. 10.1093/bioinformatics/btp666 19965881 PMC2815658

