# Supplementary figures and images for: Retrohoming of a Mobile Group II Intron in Human Cells Suggests How Eukaryotes Limit Group II Intron Proliferation
Source: PLoS Genet. 2015 Aug 4;11(8):e1005422. doi: 10.1371/journal.pgen.1005422 (PMC4524724; doi:10.1371/journal.pgen.1005422)

S1 Fig.

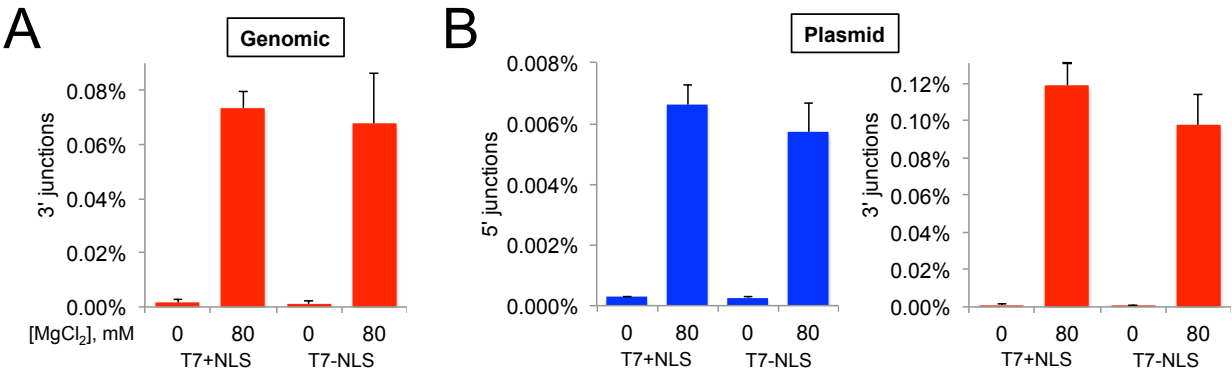

Supplement: S1 Fig — (A) Genomic and (B) plasmid retrohoming assays were done in HEK-293 in culture medium supplemented with 80 mM MgCl2 using the three-plasmid Ll.LtrB group II intron expression system with T7 RNAP expressed with or without an appended SV40 NLS. Negative controls were assays in which magnesium was not added to the cell culture medium. Retrohoming efficiencies were measured in adherent cells by the Taqman qPCR assays diagrammed in Fig 5. Blue and red bars show frequencies of 5’- and 3’-integration junctions, respectively, relative to copies of a sequence within the hygromycin-resistance gene (hyg R) adjacent to the retrohoming site. The bar graphs show the average for three separate transfections with the error bars indicating the SEM. (PDF) [file pgen.1005422.s001.pdf]

S2 Fig.

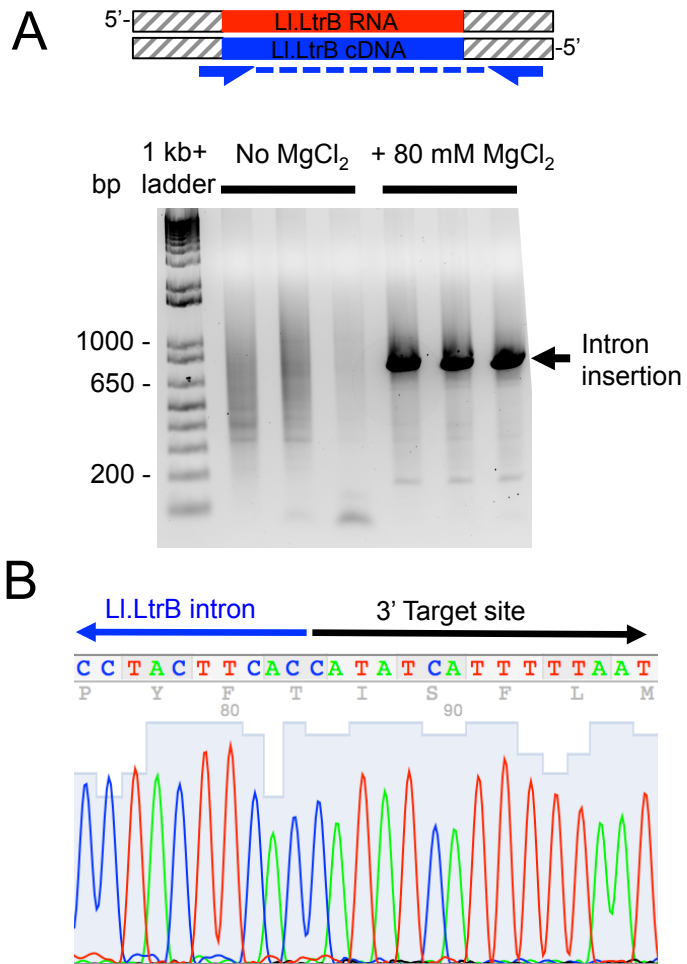

Supplement: S2 Fig — (A) PCR analysis. Retrohoming assays were done in HEK-293 Flp-In cells transfected with Ll.LtrB expression plasmids plus recipient plasmid pFRT with or without 80 mM Mg2+ added to the culture medium, as described in Fig 5. PCR was done on total DNA extracted from the cells using primers 176S 5’- CATCCATAACGTGCGCC and 269A; S3 Table). The upstream primer anneals to the 5’-integration junction (positions -10 to +7), and the downstream primer anneals to a sequence 28-nt downstream of the 3’ integration junction. Each of the samples shown is from a separate transfection. (B) Sanger sequencing of a PCR product from (A), confirming the correct 3’- integration junction. (PDF) [file pgen.1005422.s002.pdf]

S3 Fig.

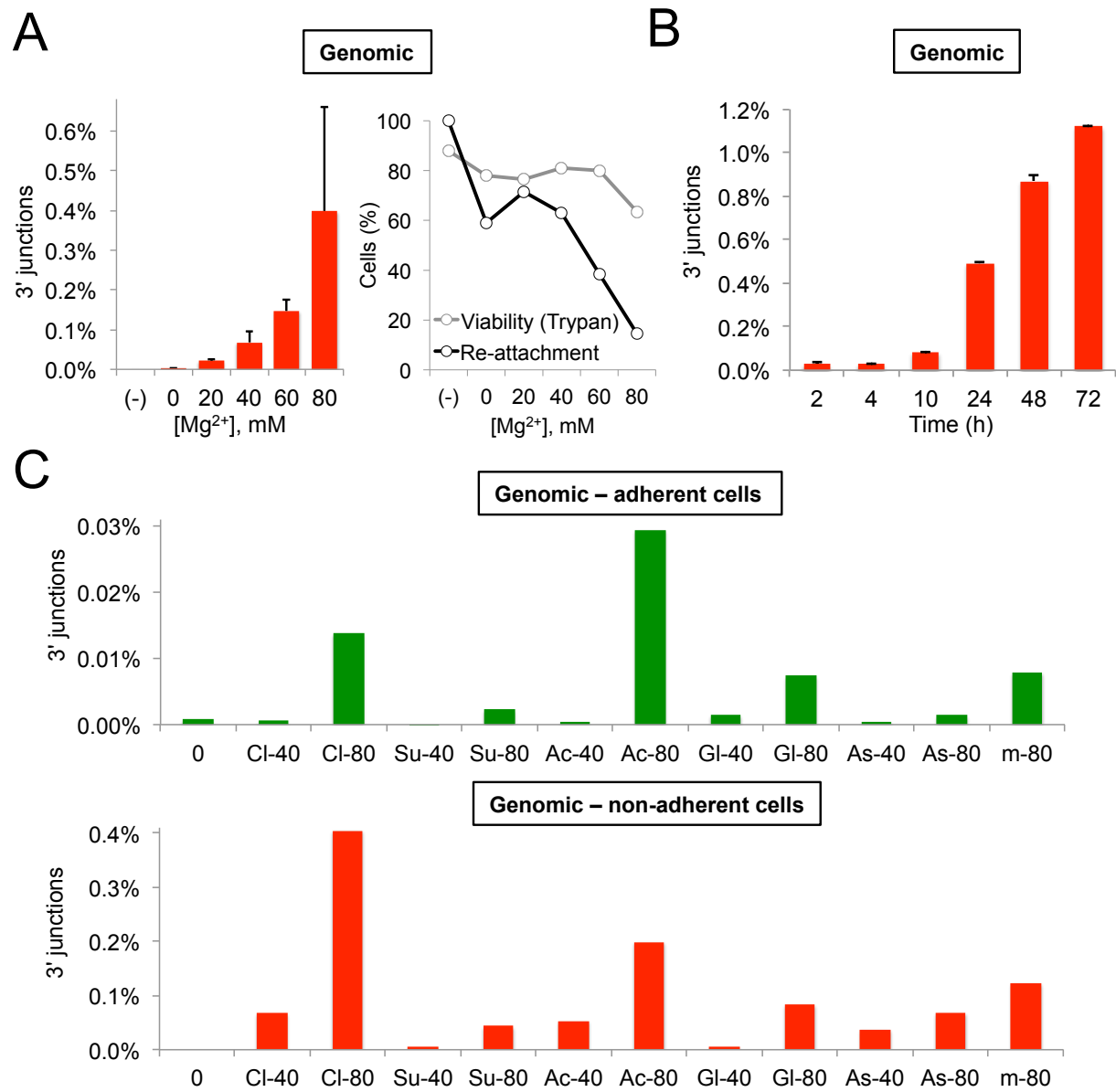

Supplement: S3 Fig — (A) Retrohoming into the genomic target site and cell viability in HEK-293 cells in culture medium supplemented with different concentrations of MgCl2. The bar graph on the left shows the retrohoming frequency assayed by Taqman qPCR of 3’-integration junctions in DNA extracted from HEK-293 cells (adherent and non-adherent) transfected with the Ll.LtrB expression plasmids after incubation in medium containing the indicated Mg2+ concentration for 24 h. The (-) control indicates absence of both phLtrA protein and MgCl2. Values are the mean for three separate transfections on the same day, with the error bar indicating the SEM. The right shows a plot of cells viability prior to DNA extraction as determined by trypan blue staining. Reattachment refers to the percentage of cells that re-adhered in 24 h after trypsin treatment and subsequent re-plating. (B) Time course for retrohoming into the genomic site in HEK-293 cells in the presence of 80 mM MgCl2. The bar graphs show retrohoming frequencies assayed by Taqman qPCR of 3’-integration junctions in DNA extracted from HEK-293 cells (adherent and non-adherent) transfected with the Ll.LtrB expression plasmids after incubation in culture medium supplemented with 80 mM Mg2+ for different times. Values are the mean for three separate transfections on the same day, with the error bars indicating the SEM. (C) Retrohoming into the genomic site in HEK-293 cells in culture medium supplemented with different Mg2+ salts. The top and bottom bar graphs compare retrohoming frequencies assayed by Taqman qPCR of 3’-integration junctions in adherent versus non-adherent HEK-293 cells after 24 h in culture medium supplemented with 40 or 80 mM of different Mg2+ salts: Cl, MgCl2; Su, MgSO4; Ac, MgOAc; Gl, Mg-glutamate; As, Mg-aspartate; and m-80, equimolar MgCl2, MgSO4, Mg-glutamate, and Mg-aspartate. The concentration used is indicated next to the counter ion abbreviation. (PDF) [file pgen.1005422.s003.pdf]

**S4 Fig.**

# A

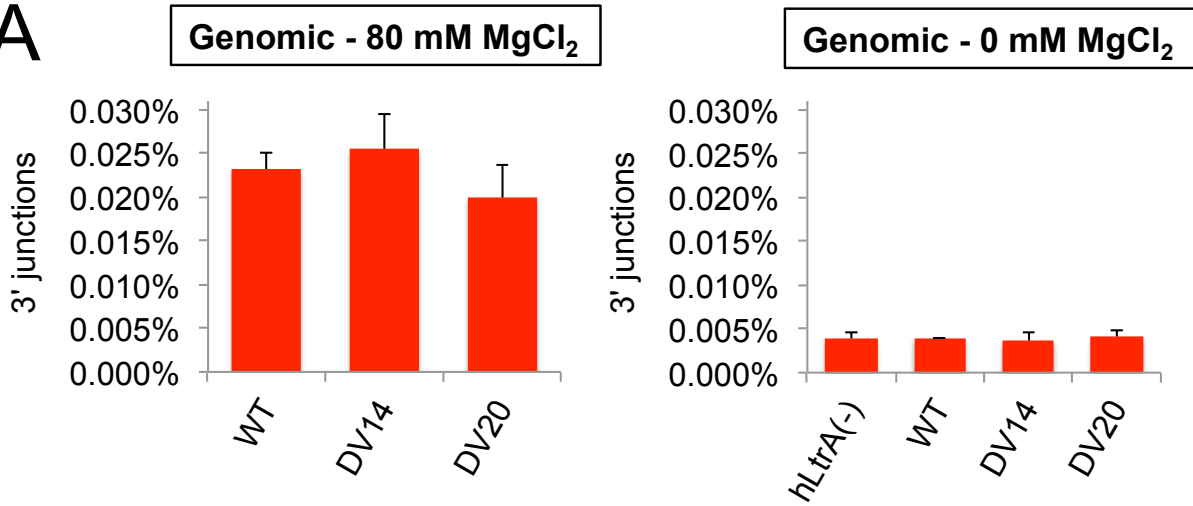

# B

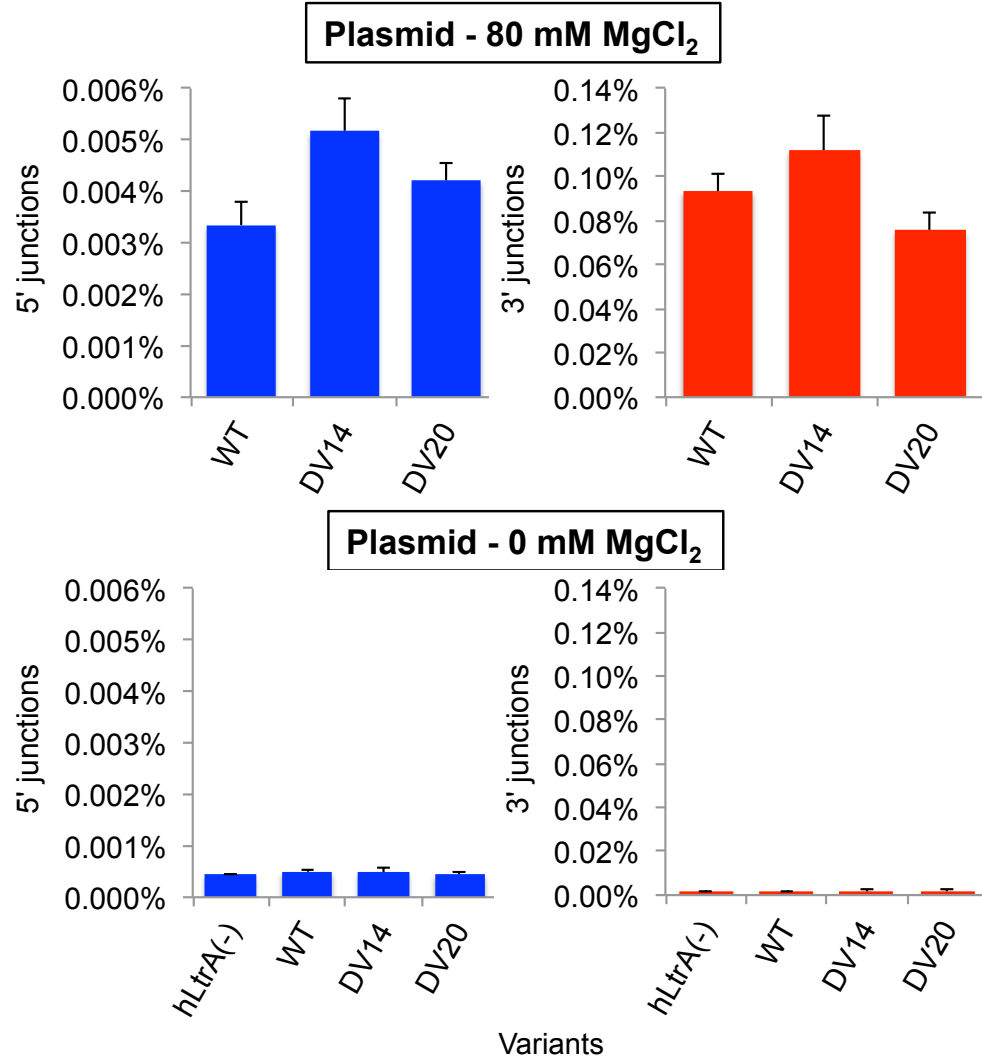

Supplement: S4 Fig — Ll.LtrB variants DV14 and DV20 with mutations in the distal stem of DV selected for enhanced retrohoming in Mg2+-deficient E. coli [36] were tested in parallel to the wild-type intron for retrohoming into (A) genomic or (B) plasmid target sites in HEK-293 cells with or without 80 mM MgCl2 added to the culture medium. Cells were transfected with phLtrA, pLl.LtrB, and pT7-NLS, and retrohoming was assayed by qPCR at 24 h after transfection. The assays done without extra Mg2+ added to the culture medium are denoted 0 mM MgCl2, and hLtrA(-) indicates a control done without transfection of phLtrA. The bar graphs show retrohoming frequencies assayed by Taqman qPCR of 5’- or 3’-integration junctions (blue and red, respectively) in adherent HEK-293 cells. Values are the mean for two or three separate transfections on the same day, with the error bars indicating the SEM. (PDF) [file pgen.1005422.s004.pdf]

**S5 Fig.**

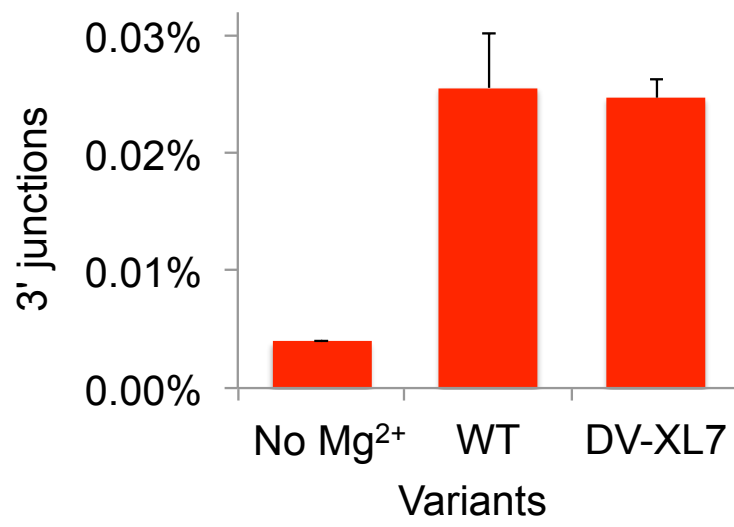

Supplement: S5 Fig — An Ll.LtrB variant (DV-XL7) with mutations in the distal stem of DV that result in four-fold increased retrohoming efficiency in Xenopus laevis oocytes [54] was tested in parallel with the wild-type intron and did not shown increased retrohoming frequencies into a genomic target site in HEK-293 cells with 80 mM MgCl2 added to the culture medium. The WT intron was tested without extra MgCl2 (No Mg2+) as a control. The bar graphs show retrohoming frequencies assayed by Taqman qPCR of 3’-integration junctions in DNA extracted from adherent HEK-293 cells transfected with the Ll.LtrB expression plasmids after incubation in medium containing the indicated Mg2+ concentration for 24 h. Values are the mean for two separate transfections on the same day, with the error bars indicating the SD. (PDF) [file pgen.1005422.s005.pdf]

S6 Fig.

A

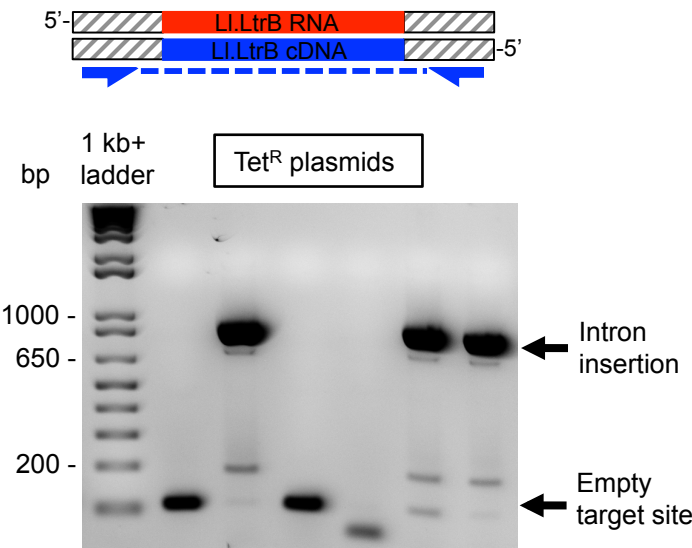

B

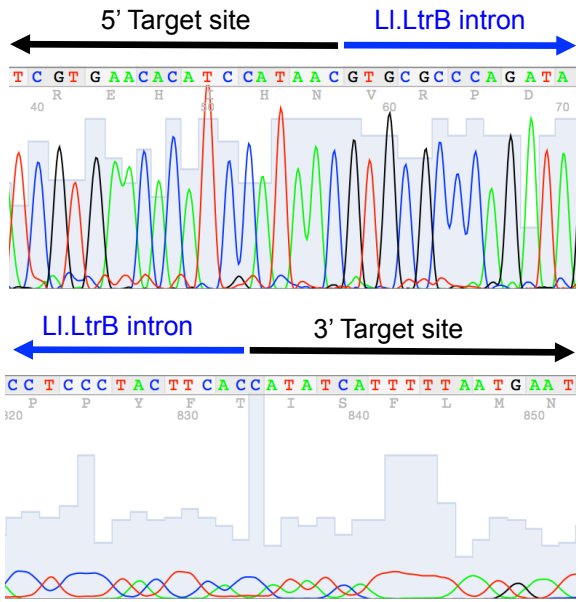

Supplement: S6 Fig — (A) PCR amplification of full-length Ll.LtrB insertions from TetR recipient plasmids recovered by selection in E. coli from HEK-293 cells after retrohoming in the presence of 80 mM MgCl2 was done using primers 200S and 269A; S3 Table). The upstream primer anneals 32-nt upstream of the integration site, and the downstream primer anneals 28-nt downstream of the integration site. Approximately 50% of recovered plasmids contain the full-length intron integrations. The remainders are false positives. (B) Sanger sequencing of full-length intron integrations from a TetR plasmid recovered by selection in E. coli, confirming the expected 5-’ and 3’- integration junctions in the same PCR product. (PDF) [file pgen.1005422.s006.pdf]

S7 Fig.

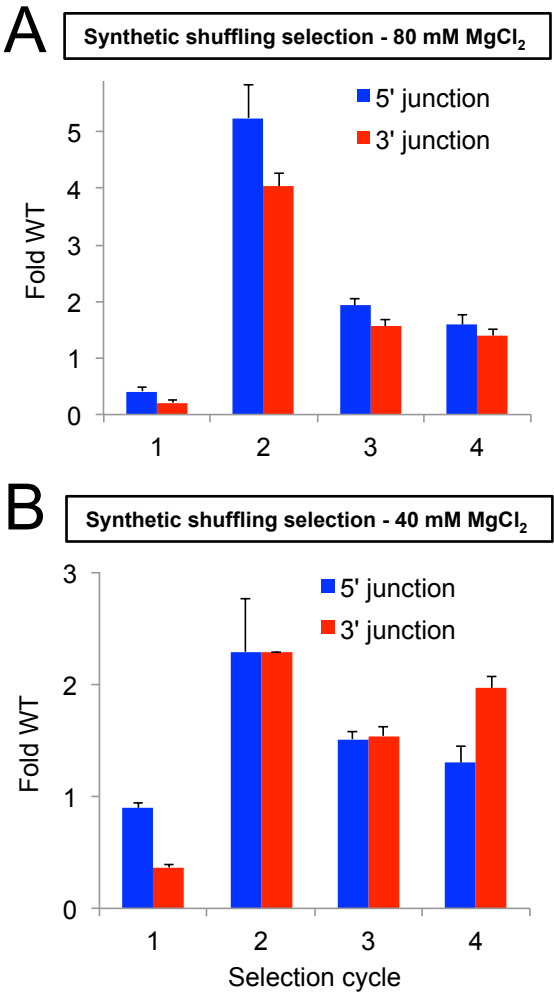

Supplement: S7 Fig — Optimal combinations of mutations were identified by synthetic shuffling and selection in HEK-293 cells for four cycles with (A) 80 mM MgCl2 or (B) 40 mM MgCl2 added to the culture medium. The synthetic shuffling library was generated as described in Fig 9. The synthetically shuffled library was tested at the indicated MgCl2 concentration, and the wild-type intron was tested in parallel. 5'- and 3'-integration junctions were quantified by Taqman qPCR relative to tet R copies during the selection cycles and expressed relative to the retrohoming frequency of the wild-type intron assayed in parallel. Values are the mean for three separate transfections on the same day, with the error bars indicating the SEM. (PDF) [file pgen.1005422.s007.pdf]
