# Supplementary material for: Retrohoming of a Mobile Group II Intron in Human Cells Suggests How Eukaryotes Limit Group II Intron Proliferation
Source: PLoS Genet. 2015 Aug 4;11(8):e1005422. doi: 10.1371/journal.pgen.1005422 (PMC4524724; doi:10.1371/journal.pgen.1005422)
Supplement: S1 Table — The frequency refers to the percentage of reads with the indicated mutations and all other positions remaining wild type after selection rounds 8 and 12. By comparison, the average frequency of variants occurring only once was ~0.03–0.07% of the total sequencing reads for each library. (DOCX) [file pgen.1005422.s008.docx]

**S1 Table.**

| **Library/number** | **Mutations** | **Frequency (%)** |
| --- | --- | --- |
| **Round 8 mutants** | | |
| hM8-1 | C622U, A643U, G651A | 0.86 |
| hM8-2 | none (wild-type) | 0.79 |
| hM8-3 | C639G | 0.72 |
| hM8-4 | U642A, G651A, U652C | 0.72 |
| hM8-5 | U642C | 0.65 |
| **Round 12 mutants** | | |
| hM12-1 | G282A, U642C | 4.99 |
| hM12-2 | U642C | 2.31 |
| hM12-3 | G282A, U642C, U661C | 1.21 |
| hM12-4 | A548C, U642C | 1.14 |
| hM12-5 | G282A, G424A, U642C | 1.07 |
