# Supplementary material for: Retrohoming of a Mobile Group II Intron in Human Cells Suggests How Eukaryotes Limit Group II Intron Proliferation
Source: PLoS Genet. 2015 Aug 4;11(8):e1005422. doi: 10.1371/journal.pgen.1005422 (PMC4524724; doi:10.1371/journal.pgen.1005422)
Supplement: S2 Table — The Table shows calculated values for standard linkage disequilibrium (D) and the normalized linkage disequilibrium (D') between the highest frequency mutations in the HEK-293 cell selection at round 8 (see Materials and Methods). The value for D and D' can be positive or negative, indicating whether the combinations of mutations occur more or less frequently, respectively, than expected from the frequency of each mutation by itself. Values close to zero indicate linkage equilibrium between the two mutations. The r 2 and Χ 2 values indicate the significance of the disequilibrium, with higher numbers indicating greater significance. (DOCX) [file pgen.1005422.s009.docx]

**S2 Table.**

| **Mutations** | ***D*** | ***D'*** | ***r^2^*** | ***Χ^2^*** |
| --- | --- | --- | --- | --- |
| G282A-A548C | -0.002 | -0.01 | 0.00016 | 0.22 |
| G282A-T642A | 0.008 | 0.12 | 0.00347 | 4.8 |
| G282A-T642C | 0.0004 | 0.01 | 0.00002 | 0.03 |
| G282A-G651A | -0.0001 | -0.001 | 0.00000 | 0.0 |
| G282A-T652C | 0.011 | 0.18 | 0.00366 | 5.1 |
| T642A-T652C | 0.088 | 1.29 | 0.41010 | 572.1 |
| T642A-G651A | 0.036 | 0.99 | 0.11108 | 155.0 |
| T642C-T652C | -0.001 | -0.10 | 0.00090 | 1.2 |
| A548C-T642A | -0.0005 | -0.01 | 0.00001 | 0.02 |
| A548C-T642C | -0.0049 | -0.07 | 0.00325 | 4.5 |
| G651A-T652C | 0.073 | 2.74 | 0.29790 | 415.6 |
| A548C-T652C | 0.0046 | 0.10 | 0.00075 | 1.1 |
| A548C-G651A | -0.0077 | -0.04 | 0.00340 | 4.7 |
