# Supplementary material for: Retrohoming of a Mobile Group II Intron in Human Cells Suggests How Eukaryotes Limit Group II Intron Proliferation
Source: PLoS Genet. 2015 Aug 4;11(8):e1005422. doi: 10.1371/journal.pgen.1005422 (PMC4524724; doi:10.1371/journal.pgen.1005422)
Supplement: S3 Table — Taqman probes and primers used for detecting retrohoming of the Ll.LtrB intron in HEK-293 cells. The hyg R target refers to the gene encoding hygromycin phosphotransferase, which confers hygromycin B resistance in the HEK-293 Flp-In cells. It is located upstream of the wild-type Ll.LtrB target site in the genomic FRT recombinase site. Taqman probes with 5'-FAM (6-carboxyfluorescien) and 3'-MGB (dihydrocyclopyrroloindole tripeptide major groove binder) were obtained from Applied Biosystems and those with 5'-FAM and 3'-BkFQ (Iowa Black FQ) from Integrated DNA Technologies. (DOCX) [file pgen.1005422.s010.docx]

**S3 Table.**

| **Target** | **Name** | **Type/**  **Orientation** | **Sequence** |
| --- | --- | --- | --- |
| 5' junction - pFRT and genomic | 198S-Q10 | Taqman probe | 5'-FAM-ATCCATAACGTGCGCCCA-MGB |
|  | 268S | Forward | 5'-CCCCAGCATGCATTACCC |
|  | 201A | Reverse | 5'- TCGGTTAGGTTGGCTGTTTTCT |
| 3' junction - pFRT and genomic | 189S-Q1 | Taqman probe | 5'-FAM-CTACTTCACCATATCATTTT-MGB |
|  | 197S | Forward | 5'-AAGAGGGTGGTGCAAACCAG |
|  | 269A | Reverse | 5'-ACGTAGATAAGTAGCATGGCGGGT |
| *hyg^R^*- pFRT and genomic | 273A | Taqman probe | 5'-FAM-AAGACCTGCCTGAAACCGAACTGCC-BkFQ |
|  | 271A | Forward | 5'-CGAGAGCCTGACCTATTGCAT |
|  | 272S | Reverse | 5-CGACCGGCTGCAGAACA |
| 5' junction - pBRRQ | 198S-Q10 | Taqman probe | 5'-FAM-ATCCATAACGTGCGCCCA-MGB |
|  | 200S | Forward | 5'-CCGCTCTAGAACTAGTGGATCCA |
|  | 201A | Reverse | 5'-TCGGTTAGGTTGGCTGTTTTCT |
| 3' junction - pBRRQ | 189S-Q1 | Taqman probe | 5'-FAM-CTACTTCACCATATCATTTT-MGB |
|  | 197S | Forward | 5'-AAGAGGGTGGTGCAAACCAG |
|  | 269A | Reverse | 5'-ACGTAGATAAGTAGCATGGCGGGT |
| *tet^R^*- pBRRQ | 338P | Taqman probe | 5'-FAM-TCGGCACCGTCACCCTGGATG-BkFQ |
|  | 336S | Forward | 5'-ACAATGCGCTCATCGTCATC |
|  | 337A | Reverse | 5'-CCGGCATAACCAAGCCTATG |
